# Boreal–Arctic wetland methane emissions modulated by warming and vegetation activity

**DOI:** 10.1038/s41558-024-01933-3

**Published:** 2024-02-14

**Authors:** Kunxiaojia Yuan, Fa Li, Gavin McNicol, Min Chen, Alison Hoyt, Sara Knox, William J. Riley, Robert Jackson, Qing Zhu

**Affiliations:** 1 Climate and Ecosystem Sciences Division, Climate Sciences Department, Lawrence Berkeley National Laboratory, Berkeley, CA USA; 2 Department of Forest and Wildlife Ecology, University of Wisconsin Madison, Madison, WI USA; 3 Department of Earth and Environmental Sciences, University of Illinois Chicago, Chicago, IL USA; 4 Department of Earth System Science, Stanford University, Stanford, CA USA; 5 Department of Geography, The University of British Columbia, Vancouver, British Columbia Canada; 6 Department of Geography, McGill University, Montreal, Quebec Canada

**Keywords:** Biogeochemistry, Climate sciences

## Abstract

Wetland methane (CH_4_) emissions over the Boreal–Arctic region are vulnerable to climate change and linked to climate feedbacks, yet understanding of their long-term dynamics remains uncertain. Here, we upscaled and analysed two decades (2002–2021) of Boreal–Arctic wetland CH_4_ emissions, representing an unprecedented compilation of eddy covariance and chamber observations. We found a robust increasing trend of CH_4_ emissions (+8.9%) with strong inter-annual variability. The majority of emission increases occurred in early summer (June and July) and were mainly driven by warming (52.3%) and ecosystem productivity (40.7%). Moreover, a 2 °C temperature anomaly in 2016 led to the highest recorded annual CH_4_ emissions (22.3 Tg CH_4_ yr^−1^) over this region, driven primarily by high emissions over Western Siberian lowlands. However, current-generation models from the Global Carbon Project failed to capture the emission magnitude and trend, and may bias the estimates in future wetland CH_4_ emission driven by amplified Boreal–Arctic warming and greening.

## Abstract (web summary)

Whether methane emissions from the Boreal–Arctic region are increasing under climate change is unclear, but critical for determining climate feedbacks. This study uses observations and machine learning to show an increase in wetland methane emissions over the past two decades, with inter-annual variation.

## Main

Methane (CH_4_) contributes approximately 20–30% of global emission-related radiative forcing^1,2^, and is the second largest source of current anthropogenic warming, with a global warming potential 28–34 times larger than that of CO_2_ over a 100-year time horizon^1,3^. Wetlands are the largest and most uncertain natural source of global CH_4_ emissions^4–6^ and wetland CH_4_ emissions are closely linked to temperature^7–9^. In a substantial portion of the Boreal–Arctic (that is, including northern boreal and tundra ecoregions and also areas north of 50° characterized by rock and ice^10,11^), recently observed warming has been occurring three to four times faster than the global average^12^, and has fuelled concerns given the positive feedbacks between CH_4_ emissions and warming^9,13,14^. However, the regional response of Boreal–Arctic wetland CH_4_ emissions to long-term environmental change remains unknown.

Warming^15^ and increasing substrate availability for soil microbes due to an observed increase in vegetation productivity^16^ should increase CH_4_ production^17,18^, all else being equal. However, warming enhances aerobic^19^ and anaerobic CH_4_ oxidation^20^, and variations in inundation areas^21,22^ could offset increased CH_4_ production. In the Boreal–Arctic region, both positive and negative trends have been reported with top-down (TD; that is, atmospheric transport inversion) and bottom-up (BU; that is, using terrestrial ecosystem models) approaches^17,18,23^ due to several sources of uncertainty, including parameterization of biogeochemical processes^17,18^, representation of atmospheric transport and photochemical sinks^6,24,25^, wetland inundation dynamics^6,21^ and limited ground observations^6,26,27^.

The magnitude of Boreal–Arctic regional wetland CH_4_ emissions also remains highly uncertain^6,24,28–31^, with previous estimates ranging from about 9 to 53 Tg CH_4_ yr^−1^ (refs. ^6,28,32–39^). Although current TD models generally agreed on higher emissions relative to BU models during 2008–2017^6^, the uncertainty ranges within both BU and TD models exceeded the magnitude of CH_4_ emissions they estimated. Notably, the uncertainty of the Boreal–Arctic wetland CH_4_ emissions is twice as large as the global atmospheric CH_4_ changes due to a sink–source imbalance of ~20 Tg CH_4_ yr^−1^ (ref. ^25^), limiting reliable conclusions on natural and anthropogenic fluxes for the global CH_4_ budget^6,24,25^.

Narrowing these substantial uncertainties in estimates of regional wetland CH_4_ emissions requires better understanding and model representations of the relationships between wetland CH_4_ emissions and environmental drivers. Previous meta-analyses have revealed a dependence of CH_4_ emissions on temperature from methanogen cultures to ecosystem scales^9^. Existing observations have also demonstrated confounding effects on CH_4_ emissions from other factors, including hydrologic and vegetation conditions^7,8,40,41^, microbial dynamics and composition^42,43^ and substrate availability^44^. Additionally, the relationships between CH_4_ emissions and environmental drivers show substantial hysteresis, hypothesized to result from time lags between primary productivity and its conversion to methanogenesis substrates^45^ and interactions between fermentation, acetate availability and acetoclastic methanogen biomass and activity^46^. These effects could largely modulate the timing and magnitude of CH_4_ emissions^7,8,13,45,47^ and affect model estimates^7,13,28^, yet they have not been explicitly considered when exploring the responses of the Boreal–Arctic wetlands to climate change.

**Fig. 1:**
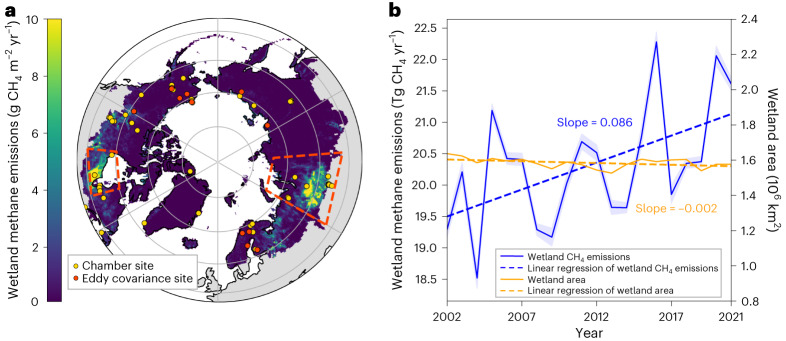
Significant increasing trend of wetland CH_4_ emissions in the Boreal–Arctic during 2002–2021. **a**, Spatial distribution of the long-term averaged wetland CH_4_ emissions in the Boreal–Arctic upscaled by combining chamber and EC datasets. Red dashed boxes indicate two wetland hotpots: WSL (52–74° N, 60–94.5° E) and HBL (50–60° N, 75–96° W). Boreal–Arctic basemap data from ref. ^72^. **b**, Annual Boreal–Arctic wetland CH_4_ emissions and Wetland Area and Dynamics for Methane Modeling (WAD2M) wetland area dataset between 2002 and 2021. Dashed lines indicate the linear regression results for wetland CH_4_ emissions (*P* = 0.017, two-sided *t*-test) and wetland area (*P* = 0.064). The blue shaded area indicates the s.d. in estimated wetland CH_4_ variability due to model parameter uncertainty.

Furthermore, Boreal–Arctic wetland CH_4_ emissions exhibit strong spatial heterogeneity^7,41,48^ and temporal variability^17,27,48^, highlighting the need for widespread flux observations to constrain models^27^. Existing eddy covariance (EC) measurements within the FLUXNET-CH_4_ network^26,27^ (Fig. 1a, red circles) over the Boreal–Arctic have been distributed over non-hotspot wetlands since 2006 (Supplementary Fig. 1a), while chamber observations (Fig. 1a, yellow circles) are available beyond EC-observed years and in wetland hotspots, that is, the Western Siberian lowlands (WSL) and Hudson Bay lowlands (HBL). Combining EC and chamber measurements thus provides expanded spatial and temporal coverage of observational constraints, albeit with challenges in reconciling the two kinds of datum with different spatial and temporal scales^27^.

Here, we quantified the decadal responses of wetland CH_4_ emissions to environmental changes in the Boreal–Arctic region by considering the lagged dependence of CH_4_ emissions on abiotic and biotic drivers and using the largest dataset of the Boreal–Arctic CH_4_ compiled to date, which combines both EC tower and chamber data (Methods). The CH_4_ emission dataset has 139 and 168 site years of EC and chamber measurements, respectively, sampled in both hotspot and non-hotspot regions (Fig. 1a). We generated an upscaled data product of Boreal–Arctic wetland CH_4_ emissions during 2002–2021 using a physically interpretable and causality-guided machine learning model^7^. Specifically, the causal relationships between CH_4_ emission and its drivers inferred from observations (Methods) were used to guide model training, achieving higher accuracy than commonly used machine learning methods^7^. Using the upscaled dataset, we investigated the predominant drivers that regulate the long-term trend and variability of CH_4_ emissions. We also benchmarked the performance of BU (*n* = 13) and TD (*n* = 21) models that participated in the most recent Global Carbon Project – CH_4_ budget^6,24^.

## Multidecadal temporal dynamics of wetland CH_4_ emissions

The upscaled Boreal–Arctic wetland CH_4_ emission dataset revealed that the mean annual emissions were 20.3 ± 0.9 (mean ± 1 s.d.) Tg CH_4_ yr^−1^ from 2002 to 2021, where ~53% of the total was contributed by the two hotspot areas (Fig. 1a, regions highlighted in the red boxes). Specifically, the largest hotspot was the WSL, which emitted 6.6 ± 0.5 Tg CH_4_ yr^−1^, ~57% larger than the second hotspot, the HBL (4.2 ± 0.3 Tg CH_4_ yr^−1^). The upscaled CH_4_ emissions were validated against randomly excluded site observations (Methods), and the Pearson correlation coefficient (*R*), mean absolute error and normalized mean absolute error between estimated and measured CH_4_ emissions were 0.89 ± 0.02, 20.81 ± 1.88 nmol CH_4_ m^−2^ s^−1^ and 3.65 ± 0.50% (Supplementary Fig. 2), respectively. Detailed information for the observation sites is found in Supplementary Tables 1 and 2.

Furthermore, a significant increasing trend (*P* < 0.05) of the Boreal–Arctic CH_4_ emissions was detected from 2002 to 2021 (Fig. 1b, blue line). The trend revealed an ~8.9% increase in CH_4_ emissions since 2002. The WSL and non-hotspot regions contributed ~56% and ~38% of the increasing trend, respectively, while no significant trend was found in the HBL (Supplementary Fig. 3). The CH_4_ emission enhancement during the boreal summer (June–August) contributed the most (~76%) to the annual-scale increasing trend (Supplementary Fig. 4), with ~62% of the increase occurring during early boreal summer (June and July). Previous observational work at a Siberian tundra site also documented a long-term increasing trend of CH_4_ emissions due to warming-induced early onset of snowmelt and vegetation growth^49^. We show here robust evidence of an increasing trend in the Boreal–Arctic region’s early summer CH_4_ emissions.

Another line of evidence for the long-term increasing trend of the Boreal–Arctic wetland CH_4_ emissions is the widespread increases in high-latitude atmospheric CH_4_ concentrations observed from the National Oceanic and Atmospheric Administration (NOAA) Global Greenhouse Gas Reference Network^50^. All high-latitude stations (18 in total, Supplementary Fig. 1b) exhibited positive trends in observed atmospheric CH_4_, and the trends of all but one station were statistically significant (*P* < 0.05) (Supplementary Table 3). Wetlands could be the dominant source of high-latitude CH_4_ emissions compared with other sources^6^, particularly in the boreal summer months^51^. The increases in atmospheric CH_4_ concentrations therefore probably reflect the increases in CH_4_ emissions from wetlands.

## Drivers of wetland CH_4_ emission variability and trend

**Fig. 2:**
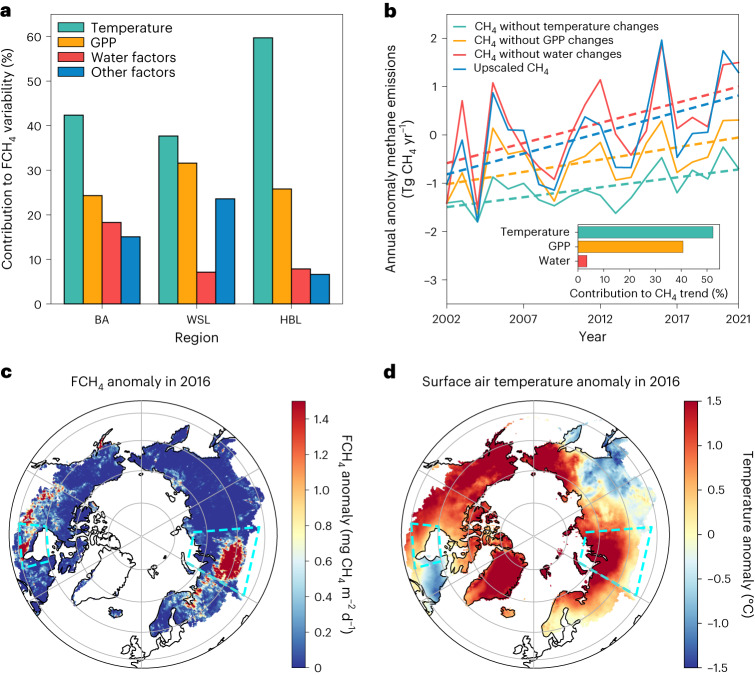
Temperature dominated the variability and trend of wetland CH_4_ emissions, and triggered the peak CH_4_ emissions in 2016. **a**, Contribution of abiotic and biotic drivers to wetland CH_4_ flux (FCH_4_) variability in the Boreal–Arctic (BA), WSL and HBL, represented as the percentage of grids where wetland FCH_4_ variability is dominated by temperature, GPP, water-related drivers (soil moisture content, and precipitation) and other drivers. **b**, Upscaled CH_4_ emission annual anomalies (solid lines) and trends (dashed lines) using all drivers and iteratively excluding the temporal dynamics for each group of drivers. Inset: contributions of different drivers to the CH_4_ emission trend (Methods). **c**,**d**, CH_4_ emission anomalies calculated relative to the multiyear annual-mean CH_4_ emissions from 2002 to 2021: anomaly of FCH_4_ (**c**) and surface air temperature^73^ (**d**) in the peak emission year of 2016. The regions marked with dashed boxes are two wetland hotpots: WSL and HBL.

Since no long-term increasing changes in wetland area were found over the Boreal–Arctic region^21^ during the past two decades (Fig. 1b, yellow line), our results suggested that the increasing trend of regional wetland CH_4_ emissions was induced primarily by changes in CH_4_ emission intensity rather than expansion of total wetland area. After accounting for confounding effects from other abiotic and biotic factors (Methods), temperature was identified as the predominant control on wetland CH_4_ emission variability over the Boreal–Arctic (Fig. 2b). Specifically, temperature dominated the variability in most grid cells (42.4%), followed by gross primary productivity (GPP) (24.3%), while water-related factors (soil water content, and precipitation) dominated the other 18.3% of grid cells. Consistent patterns of the predominant drivers were also found in the two wetland hotspots, and the grid cells with wetland CH_4_ flux observations. About 37.7%, 59.7% and 61.3% of grid cells were dominated by temperature in the WSL, HBL and the full observation-covered area, respectively (Fig. 2a and Supplementary Fig. 5). Temperature is closely linked to wetland CH_4_ production and emissions, while GPP could be a proxy for substrate availability and plant-mediated CH_4_ transport^7,8,47^. The dominance of temperature and GPP effects is consistent with previous studies^7,8,41,47^, implying a potential sensitivity of wetland CH_4_ emissions to warming and vegetation activities^52^.

For potential drivers of the Boreal–Arctic wetland CH_4_ emission trend, we used a statistical model (Methods)^53,54^ to partition the contributions from various factors including temperature, vegetation activities and water conditions. We found that in the Boreal–Arctic (Fig. 2b) temperature explained 52.3% of the increasing CH_4_ emission trend, followed by GPP (40.7%). In the Boreal–Arctic, significant increases in temperature^12^ and GPP^52,55^ have been detected using satellite-based products. While warming can increase both wetland CH_4_ production and oxidation in observations^19,20^ and model simulations^56^, our analysis here revealed net positive effects of temperature on CH_4_ emissions. Meanwhile, warming has also increased vegetation productivity in the Boreal–Arctic^52^, which could promote plant-mediated CH_4_ transport via aerenchyma tissue and increase organic substrate supply for microbes. The increase of substrate availability could fuel methanogens^57–59^, leading to an increase in wetland CH_4_ emissions^49^.

Given the dominant control of temperature on wetland CH_4_ emissions, high CH_4_ emissions can be triggered by abnormally high temperatures over high-emission wetlands. Anomalously high (2005, 2016 and 2020) and low (2004, 2009 and 2014) CH_4_ emission years (Fig. 2b) had higher and lower annual-mean temperatures, respectively, particularly in the two CH_4_ hotspot regions (Supplementary Figs. 6 and 7). The highest-emission year occurred in 2016 (Fig. 2c,d), which was the warmest year in the high latitudes since 1950^12^. The anomalously high temperature in 2016 was suggested to be caused by the major El Niño event during 2015–2016^60,61^. This strong El Niño event changed large-scale divergence and convergence patterns and upper-level moisture transport^62^, leading to subsequent changes in adiabatic warming over the Arctic surface^61,63^. The resultant high temperature happened to overlap with wetland hotspots (Fig. 2d) and induced a sharp increase (~15.5% higher emissions relative to 2002) in wetland CH_4_ emissions, particularly over the WSL (Fig. 2c). All sites except one (covering 2016 and its adjacent years) agreed with anomalously high wetland CH_4_ emissions in 2016 when the temperature was anomalously high (Supplementary Table 4). These results highlight the role of major El Niño–Southern Oscillation events in driving wetland CH_4_ emission variability^64–66^, and demonstrate a critical ecological teleconnection from the sea surface temperature of the tropical Pacific to the Boreal–Arctic wetland CH_4_ emissions.

## Implications for modelling wetland CH_4_ emissions

**Fig. 3:**
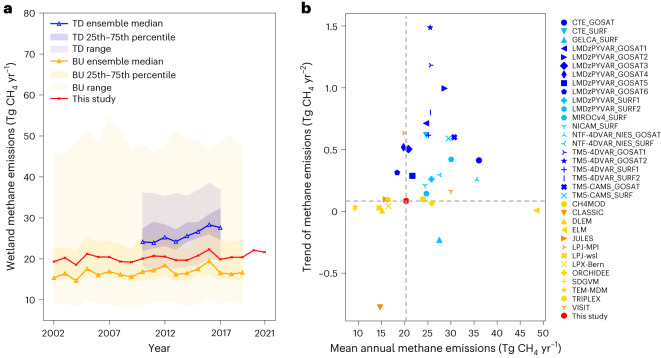
BU and TD models exhibited substantial uncertainties in the magnitude and trend of wetland CH_4_ emissions over the Boreal–Arctic region. **a**, The multimodel ensemble median (50th percentile) of wetland CH_4_ emissions estimated by TD (blue line) and BU (orange line) models, compared with the upscaled wetland CH_4_ emissions (red line). The darker shaded areas represent 25th to 75th percentiles and the lighter shaded areas represent the ranges of wetland CH_4_ emissions. **b**, The mean annual magnitudes and trends of wetland CH_4_ emissions estimated by TD and BU models, compared with those of the upscaled wetland CH_4_ emissions (red circle). For TD models, dark-blue markers indicate that the trends are significant, that is, *P* < 0.05, and light-blue markers indicate that the trends are not significant, that is, *P* > 0.05. For BU models, orange markers indicate that the trends are significant, and yellow markers indicate that the trends are not significant. The *P* values can be seen in Supplementary Table 5, obtained from a two-sided *t*-test.

Most of the current generation of BU and TD models in the Global Carbon Project CH_4_ budget^6^ did not capture the observed magnitude and trend of wetland CH_4_ emissions in the Boreal–Arctic (Fig. 3a,b). For emission magnitude, 19 out of 21 TD models overestimated and 9 out of 13 BU models underestimated the Boreal–Arctic wetland CH_4_ emissions when compared with our upscaled dataset (Fig. 3b, Supplementary Fig. 8 and Supplementary Table 5). The rest (4 of 13) of the BU models overestimated the Boreal–Arctic wetland CH_4_ emissions by 18% to 139%, with the ensemble median of all BU models (16.66 Tg CH_4_ yr^−1^) lower than that of the observationally constrained upscaled dataset (Fig. 3a). For the long-term trend, the majority (10 of 13) of BU models did not show the significantly increasing trend, while the other BU models with increasing trends differed by up to sixfold in trend magnitude relative to the upscaled trend. More (12 of 21) TD models exhibited significant increasing trends, but the trend magnitude differed by a factor ranging from 2 to 16 (Supplementary Table 5).

The lack of increasing trends in most of the current-generation process-based biogeochemical models suggests probable underestimation of future warming-induced increases in wetland CH_4_ emissions. Future Boreal–Arctic warming could further increase the intensity of wetland CH_4_ emissions and stimulate a large increase in wetland extent due to permafrost thaw^14^ and greater precipitation^67^. The underestimated increasing trend indicates that the BU model underestimated intensity in wetland emissions rather than wetland extent since all models prescribed the same wetland extent data^21^ with no significant temporal changes in wetland area (Fig. 1b). The underestimated intensity of CH_4_ emissions therefore could be multiplied by future expanded wetland area, leading to amplified underestimation of wetland CH_4_ emissions and their positive feedbacks with warming. The upscaling models showed good performance and confirmed the increasing wetland CH_4_ emission trend in the Boreal–Arctic during 2002–2021, even considering the uncertainties from which site observations were used and validation schemes (Supplementary Fig. 9), wetland extent (Supplementary Fig. 10a) and input variables (Supplementary Fig. 10b). The increasing wetland CH_4_ emissions in the high latitudes indicate the growingly important role of biogenic CH_4_ emissions in rising atmospheric CH_4_^68–70^. Underrating the upward trend of wetland CH_4_ emissions, therefore, suggests underestimated biogenic contributions to observed increases in atmospheric CH_4_ and radiative forcing^25^.

Our data-driven, long-term and spatially explicit wetland CH_4_ emission dataset opens up new opportunities to better understand the dynamics of the Boreal–Arctic wetland CH_4_ emissions and could facilitate the improvement of BU and TD models. The upscaled dataset is well constrained by widespread observations, particularly during the summer season (Supplementary Tables 1 and 2) when the major increasing trend of wetland CH_4_ emissions was reported. The dataset also explicitly considers the frequently acknowledged but underrepresented hysteresis characteristics in wetland CH_4_ emissions^7,8,13^. We demonstrated the dominant controls of temperature and GPP on the CH_4_ emission trend and variability, suggesting the priority of refining CH_4_ emission temperature sensitivity and plant-modulated CH_4_ emission processes in BU models. For example, the temperature dependence of CH_4_ emissions has been empirically represented and poorly parameterized in biogeochemical models^13^. Plant-mediated microbial substrate availability is strongly linked to CH_4_ emissions^57^, yet has rarely been mechanistically represented in models^13^. Current biogeochemical models have little consensus on model structure or complexity in representing wetland CH_4_ emissions^71^. Confidence in model predictions is limited by knowledge gaps and ability to validate models across time and space. This new benchmark dataset could aid validation and parameterizations of the highly uncertain biogeochemical processes related to CH_4_ emissions. Additionally, the upscaled dataset provides better prior information for TD transport inversion models, thereby allowing for more reliable separation of natural and anthropogenic CH_4_ emission effects on atmospheric CH_4_ concentrations.

## Methods

### Wetland datasets

We used the WAD2M dataset^21^ derived from active and passive microwave remote sensing as the prescribed wetland extent. When compared with the optical-sensor-based products, the microwave-sensor-based WAD2M product can identify water conditions below vegetation canopies; the product also mitigated the risks of double counting wetland and water bodies in the Boreal–Arctic region by fusing multisource wetland extent datasets^21^. A promising capability of the WAD2M dataset is its ability to capture the inter-annual variations of wetland extent^21^. In addition to inundation dynamics, clear differences in the magnitude and processes of wetland CH_4_ emissions have been reported across different wetland types^7,8,41,47^. Therefore, wetland types extracted from the Boreal–Arctic Wetland and Lake Dataset (BAWLD)^11^ were used to separately model wetland CH_4_ emissions across bogs, fens, marshes and tundra. The BAWLD dataset also avoided the double-counting problem^11^. All wetland grid cells in the BAWLD dataset that provided the wetland type information were considered for upscaling, and the wetland type percentage provided by the BAWLD dataset was used for partitioning the wetland extent of the corresponding grid cells in the WAD2M dataset for each wetland type (Supplementary Section 1).

In addition, we also used other wetland datasets, including static wetlands from the Global Lakes and Wetlands Database^74^, and temporally dynamic model-derived wetlands^75^ calibrated by observations from Global Inundation Extent from Multi-Satellites^76^ and Regularly Flooded Wetland^77^. We discussed the temporal changes of wetland CH_4_ emissions in the Boreal–Arctic during 2002–2021 related to the uncertain wetland extent. More details of the three wetland datasets used and the sensitivity experiments are given in Supplementary Section 3.

### Input datasets

Temperature-, plant- and water-related variables that have been shown to be important for explaining wetland CH_4_ dynamics were used as input drivers for upscaling^7,8,41,47^. Specifically, the input variables include soil temperature (TS), air temperature (TA), GPP, air pressure (PA), precipitation (P), wind speed (WS), snow cover (SC) and soil water content (SWC). GPP was obtained from the GOSIF dataset^78^, which was derived on the basis of solar-induced chlorophyll fluorescence (SIF) observed with the Orbiting Carbon Observatory-2 and its linear relationship with GPP^79,80^. Other variables were obtained from the land component of the fifth generation of European Reanalysis (ERA5-Land) datasets^73^ because of the high accuracy and physical consistency among different variables^73,81,82^. All variables were unified to the same temporal (7 d) and spatial (0.5°) resolution, and the final upscaled dataset had the same spatiotemporal resolution as the inputting variables.

Additionally, we also used other sources of input datasets to assess the sensitivity of the temporal changes of wetland CH_4_ emissions in the Boreal–Arctic during 2002–2021 to uncertainties in the input datasets. These additional datasets included University of East Anglia Climatic Research Unit Japanese Reanalysis^83^, Global Land Data Assimilation System^84^, Modern-Era Retrospective Analysis for Research and Applications v.2 ^85^ and Penman–Monteith–Leuning GPP^86^ datasets. More details of the datasets used and the sensitivity experiments are given in Supplementary Section 4.

### Wetland CH_4_ emission observations

Substantial spatial heterogeneity of wetland CH_4_ emissions has been highlighted in the literature^7,41,48^, and therefore sparse observations may impede reliable upscaling. To overcome this issue of spatial heterogeneity, we first compiled a comprehensive CH_4_ dataset that broadly covered the Boreal–Arctic region, by combining the FLUXNET-CH4 dataset^27^, the BAWLD-CH4 dataset^28^ and the chamber dataset in ref. ^48^. We selected all chamber sites that explicitly included the wetland types considered here and start and end months of the observations^28,48^. We used quality-assured observed wetland CH_4_ emissions at EC sites instead of gap-filled data. In total, this study included 139 and 168 site years of EC and chamber measurements, respectively. Detailed information (including site identifier, wetland type, location, temporal coverage, digital object identifier and references) on the sites is listed in Supplementary Tables 1 and 2. The compiled and upscaled dataset will be made available upon reasonable request.

### Causality-guided machine learning (Causal-ML) upscaling

A Causal-ML model^7^ with good physical interpretability and accuracy was used for upscaling the wetland CH_4_ emissions. The model first identified the causal relationships between each driver and CH_4_ emission by excluding the confounding effects from other drivers through a PCMCI method^7,87–90^ (PC refers to the model inventors, P. Spirtes and C. Glymour^91^, and MCI is the acronym for momentary conditional independence^90^). The PCMCI method has been frequently used in Earth science^88,90,92–95^, and is particularly suitable for inferring multivariate controlled and time-lagged causal relationships^90,93–95^, such as those between wetland CH_4_ emission and its drivers^7,8,47^ (see Supplementary Section 2 for more details of the causality inference). Then, the identified causal structures along with the model biases between observations and model simulations were used to guide model training. This modelling strategy helps reduce model biases and improve model physics^7^. Another benefit of this Causal-ML model is the representation of time-lagged controls, which has been shown to be important for understating and modelling wetland CH_4_ dynamics^7,13^. Here, we considered the substantial intra-seasonal hysteresis found within wetland CH_4_ emissions^13^, and differentiated the wetland-type-dependent CH_4_ emission processes by building Causal-ML models for each wetland type. We randomly sampled 10% of site observations that the Causal-ML model had never seen as the testing dataset, and used the remaining 80% and 10% of the dataset to train and validate the model^7^, respectively. Through each experiment including data sampling and model training, we derived a Causal-ML model, and we repeated the experiments and upscaled the wetland CH_4_ emission dataset 20 times. The ensemble mean of the 20 upscaled datasets was used to analyse the wetland CH_4_ dynamics, and the s.d. was considered as the upscaling uncertainty related to trained model parameters caused by random data sampling. In addition, we also used the leave-one-out and temporal-cross-validation schemes for model evaluation and upscaling. For the leave-one-out scheme, we iteratively removed data from each site, retrained the model and then evaluated model performance^7^. For the temporal-cross-validation scheme, we used 20% and 80% of temporally continuous data for each site as the testing and training datasets, respectively. With the well trained models from the two additional validation schemes, we upscaled the wetland CH_4_ emissions during 2002–2021. For high-frequency (that is, weekly, daily and hourly) measurements, the errors between modelled and observed wetland CH_4_ emissions at the weekly scale were used in the objective function to direct the model training; for low-frequency (for example, some chamber observations only provided seasonal or annual-mean) measurements, the mean values of modelled and measured wetland CH_4_ emissions during the observation period were compared and used in the objective function. Details of the model parameter settings, model training and validation are given in our previous work^7^.

### Identifying dominant controls on wetland CH_4_ variability

To separate dominant controls on the inter-annual variations of wetland CH_4_ emissions, we used a simple method of partial correlation^92^. We conducted analyses between CH_4_ emission intensity and all input variables at the annual scale for each grid cell. Before the partial correlation analysis, the annual anomaly of each variable was derived by subtracting the long-term (2002–2021) annual mean and removing the inter-annual trend^92^. For each grid cell, the driver with the highest magnitude (absolute value) of partial correlation coefficient was determined as the dominant driver. Finally, we classified all the drivers into four groups, including temperature (TS and TA), GPP, water-related factors (P and SWC) and others (WS, PA and SC). We acknowledge that the dependence of wetland CH_4_ emissions on environmental predictors could vary across spatiotemporal scales^8,49,96^. Here we mainly focused on the dynamics of wetland CH_4_ emissions at the inter-annual scale. At this scale, we found strong relationships between CH_4_ emissions and environmental variables related to temperature, water and vegetation, consistent with previous studies^27,49^. We also acknowledge that these environmental variable dependences could vary over space, and become weaker when the dominant factors are beyond those considered in this study^96^. Due to spatial heterogeneity, we reported the dominant controls on the basis of the summary statistics of all wetland grid cells in the studied region with the CH_4_ dynamics significantly explained by the considered environmental factors.

### Quantifying dominant controls on wetland CH_4_ emission trend

Following previous studies^97,98^, a statistical linear regression model was used to quantify the controls from different drivers on the trend of wetland CH_4_ emissions. In particular, we first built a linear model driven by all temporally changing input factors denoted Model_all_, to quantify the responses of wetland CH_4_ emissions to environmental changes for each wetland grid cell. Then we iteratively held one group of factors, including temperature, GPP and water-related factors, constant at the corresponding initial level while allowing the other factors to change over time. For example, we used Model_*T*_ to represent the model results that held temperature constant at 2002 values and allowed all the other factors to change over time. Similarly, we used Model_GPP_ and Model_water_ to represent the model results that kept GPP and water-related variables constant, respectively, while other factors varied over time. The differences ∆CH_4_(*T*), ∆CH_4_(GPP) and ∆CH_4_(Water) (equations (1)–(3)) were regarded as the impacts of changes in temperature, GPP and water-related variables on wetland CH_4_ emission changes, respectively. The trend differences were regarded as the contributions from each group of factors to the increasing trend in wetland CH_4_ emissions^97,98^. The model parameters (that is, the slope and intercept) for each grid cell were obtained by minimizing the sum of ordinary least squares of the errors^53,54,99^.

1 $$\Delta {\mathrm{CH}}_{4}(T\,)={\mathrm{Model}}_{\mathrm{all}}-{\mathrm{Model}}_{T}={\beta }_{T}\,\Delta T$$

2 $$\Delta {\mathrm{CH}}_{4}({\mathrm{GPP}})={\mathrm{Model}}_{\mathrm{all}}-{\mathrm{Model}}_{\mathrm{GPP}}={\beta }_{\mathrm{GPP}}\,\Delta {\mathrm{GPP}}$$

3 $$\Delta {\mathrm{CH}}_{4}({\mathrm{Water}})={\mathrm{Model}}_{\mathrm{all}}-{\mathrm{Model}}_{\mathrm{water}}={\beta }_{\mathrm{water}}\,\Delta {\mathrm{Water}}.$$

## Online content

Any methods, additional references, Nature Portfolio reporting summaries, source data, extended data, supplementary information, acknowledgements, peer review information; details of author contributions and competing interests; and statements of data and code availability are available at 10.1038/s41558-024-01933-3.

### Supplementary information

Supplementary Information Supplementary Figs. 1–10, Tables 1–5 and Sections 1–4.

## Data Availability

Data are available from the following sites: WAD2M, https://zenodo.org/records/3998454; BAWLD, 10.18739/A2C824F9X (ref. ^72^); GOSIF, https://globalecology.unh.edu/data/GOSIF-GPP.html; ERA5-Land, https://www.ecmwf.int/en/forecasts/datasets/reanalysis-datasets/era5.

## Code availability

The code used in this study is available at ref. ^100^.
