# Supplementary material for: Boreal–Arctic wetland methane emissions modulated by warming and vegetation activity
Source: Nat Clim Chang. 2024 Feb 14;14(3):282–8. doi: 10.1038/s41558-024-01933-3 (PMC10927558; doi:10.1038/s41558-024-01933-3)
Supplement: Supplementary file 1 — Supplementary Figs. 1–10, Tables 1–5 and Sections 1–4. [file 41558_2024_1933_MOESM1_ESM.pdf]

nature climate change

Article

https://doi.org/10.1038/s41558-024-01933-3

Boreal–Arctic wetland methane emissions modulated by warming and vegetation activity

In the format provided by the authors and unedited

Table of contents

Figure S1. Temporal coverage of chamber and eddy covariance observations, and the locations of atmospheric methane content observation sites.
Figure S2. Observed versus model simulated wetland methane emissions.
Figure S3. Yearly total wetland CH_4 emissions from 2002 to 2021.
Figure S4. Wetland CH_4 emissions in the Boreal-Arctic area from 2002-2021 during each season.
Figure S5. Contribution of abiotic and biotic drivers to wetland CH_4 emissions.
Figure S6. Wetland methane emission anomaly, and surface air temperature anomaly in 2005 and 2020.
Figure S7. Wetland methane emission anomaly, and surface air temperature anomaly in 2004, 2009, and 2014.
Figure S8. Inter-annual wetland CH_4 emissions in the Boreal-Arctic.
Figure S9. Model performance and increasing trend of wetland CH_4 emissions in the Boreal-Arctic during 2002-2021 using the leave-one-out validation and the temporal-cross-validation schemes.
Figure S10. Wetland CH_4 emissions with increasing trends in the Boreal-Arctic during 2002-2021 using different wetland extent datasets and different forcing datasets.
Table S1. Information of eddy covariance sites in this analysis.
Table S2. Information of chamber sites used to constrain the model in this study.
Table S3. Site id, name, and location of the atmospheric methane content observation sites.
Table S4. Sites that cover the year 2016 and its adjacent years, and show anomaly high temperature in 2016.
Table S5. Annual wetland methane emissions and trends estimated by different models.
Text S1. Combination of the BAWLD and WAD2M datasets.
Text S2. Inferring causal relationships.
Text S3. Sensitivity of CH_4 dynamics to wetland extent datasets.
Text S4. Sensitivity of CH_4 dynamics to inputting datasets.

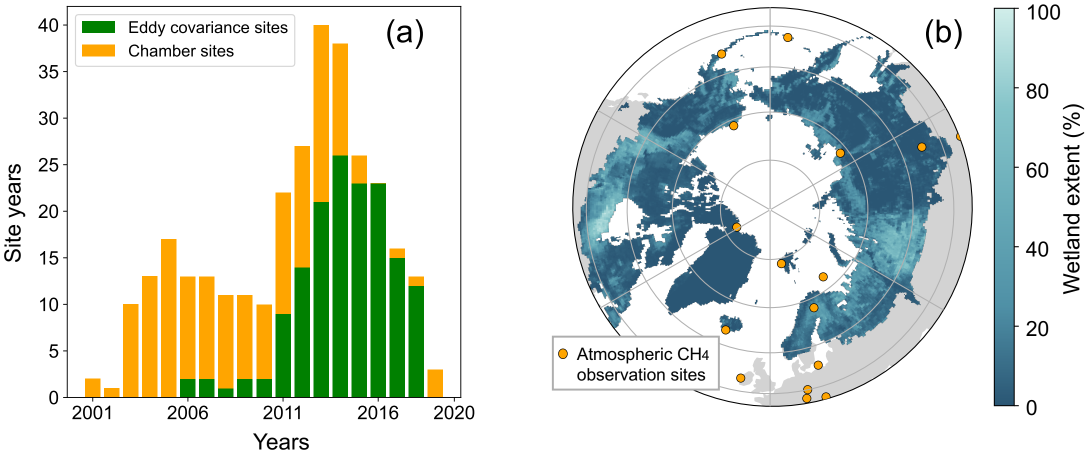

Fig. S1. (a) Temporal coverage of chamber and eddy covariance observations, and (b) the locations of atmospheric methane content observation sites.

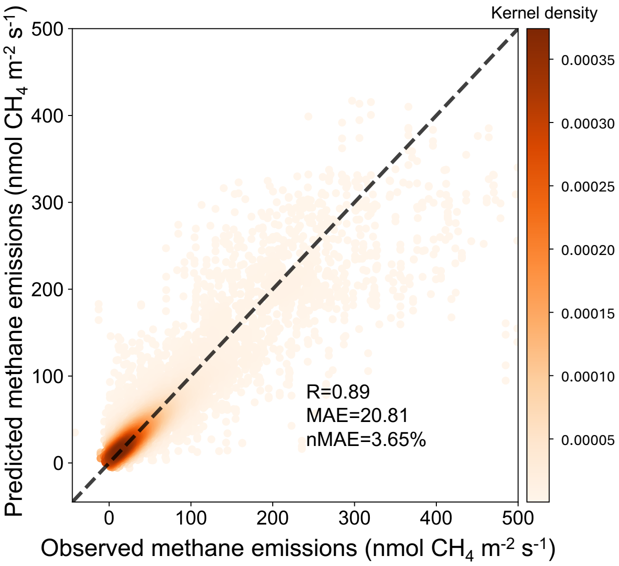

Fig. S2. Observed versus model simulated wetland methane emissions. Validation results showed that the Pearson correlation coefficient (R), Mean Absolute Error (MAE), and normalized MAE (nMAE) between estimated and measured CH_4 emissions were 0.89, 20.81 (nmol CH_4 m^{-2} s^{-1}), and 3.65%, respectively.

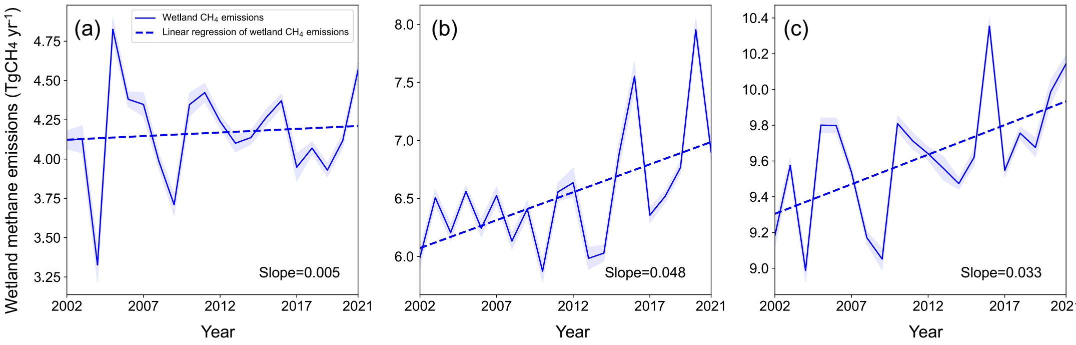

Fig. S3. Yearly total wetland CH_4 emissions (solid lines) from 2002 to 2021 and linear regression results (dashed lines) in (a) the Hudson Bay lowlands (p=0.718, two-sided t-test), (b) the Western Siberian lowlands (p=0.012), and (c) the rest of the Boreal-Arctic area excluding hotspots (p=0.009). The shaded blue area indicates the standard deviation in estimated wetland CH_4 variability due to model parameter uncertainty.

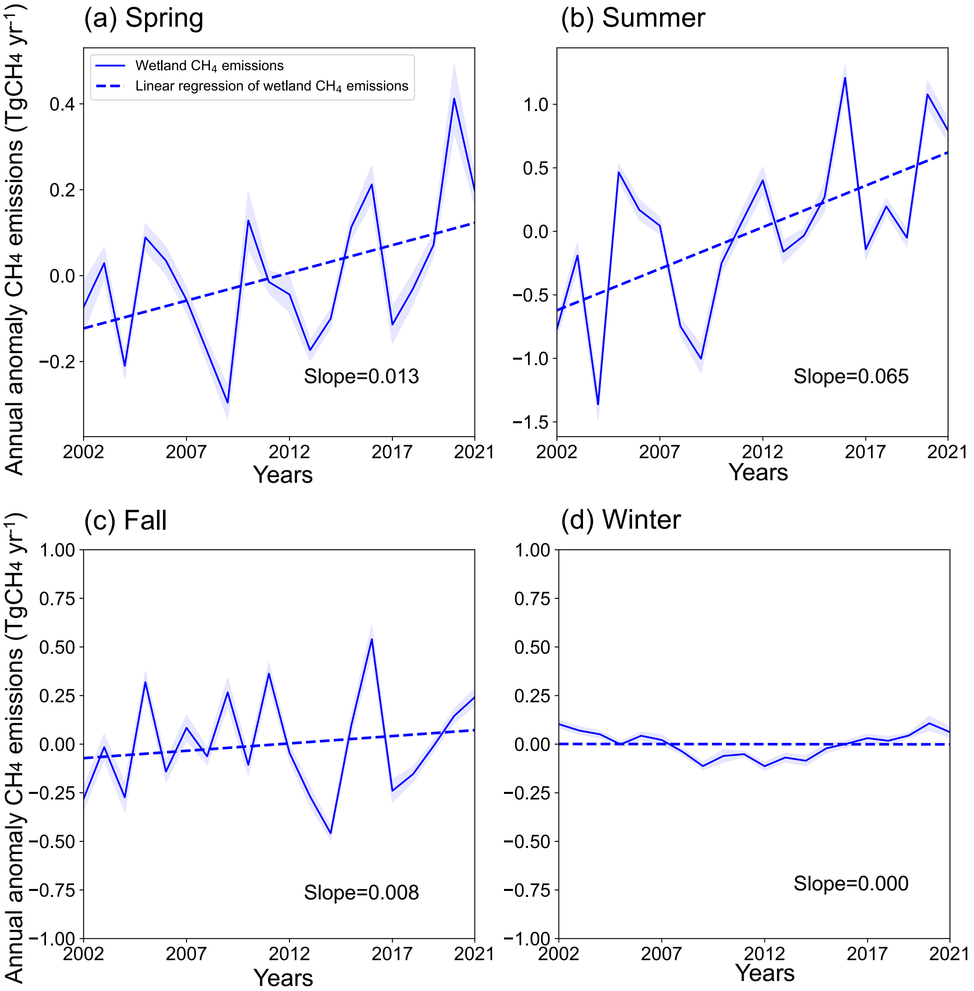

Fig. S4. Wetland CH_4 emissions in the Boreal-Arctic area from 2002-2021 during each season. The shaded blue area indicates the standard deviation in estimated wetland CH_4 emissions due to model parameter uncertainty; the dashed blue lines indicate the linearly regressed trends of wetland CH_4 emissions.

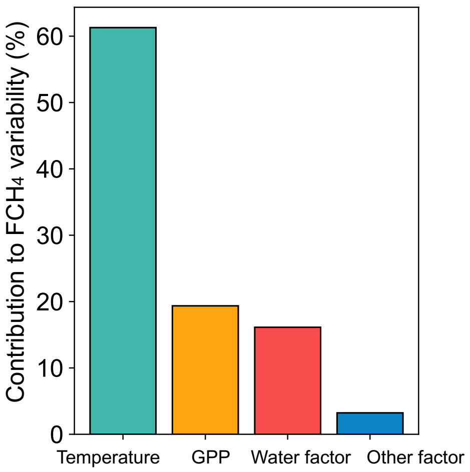

Fig. S5. Contribution of abiotic and biotic drivers to wetland CH_4 emissions (FCH_4) variability in the grid cells with site observations, represented as the percentage of grids where wetland FCH_4 variability is dominated by temperature, GPP, water-related drivers (soil moisture content and precipitation), and other drivers.

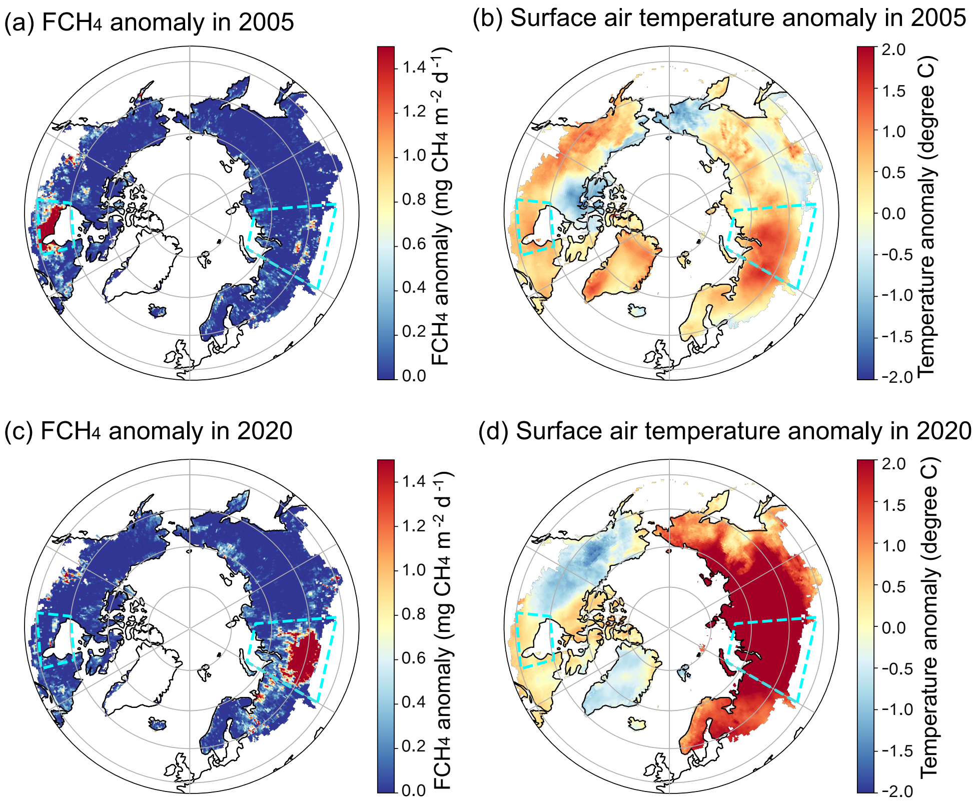

Fig. S6. Wetland methane flux (FCH_4) anomaly, and surface air temperature anomaly in 2005 and 2020. The anomalies were calculated relative to the multi-year annual mean value from 2002 to 2021. The dashed boxes marked regions in (a)-(d) are two wetland hotpots: Western Siberian lowland and Hudson Bay lowland.

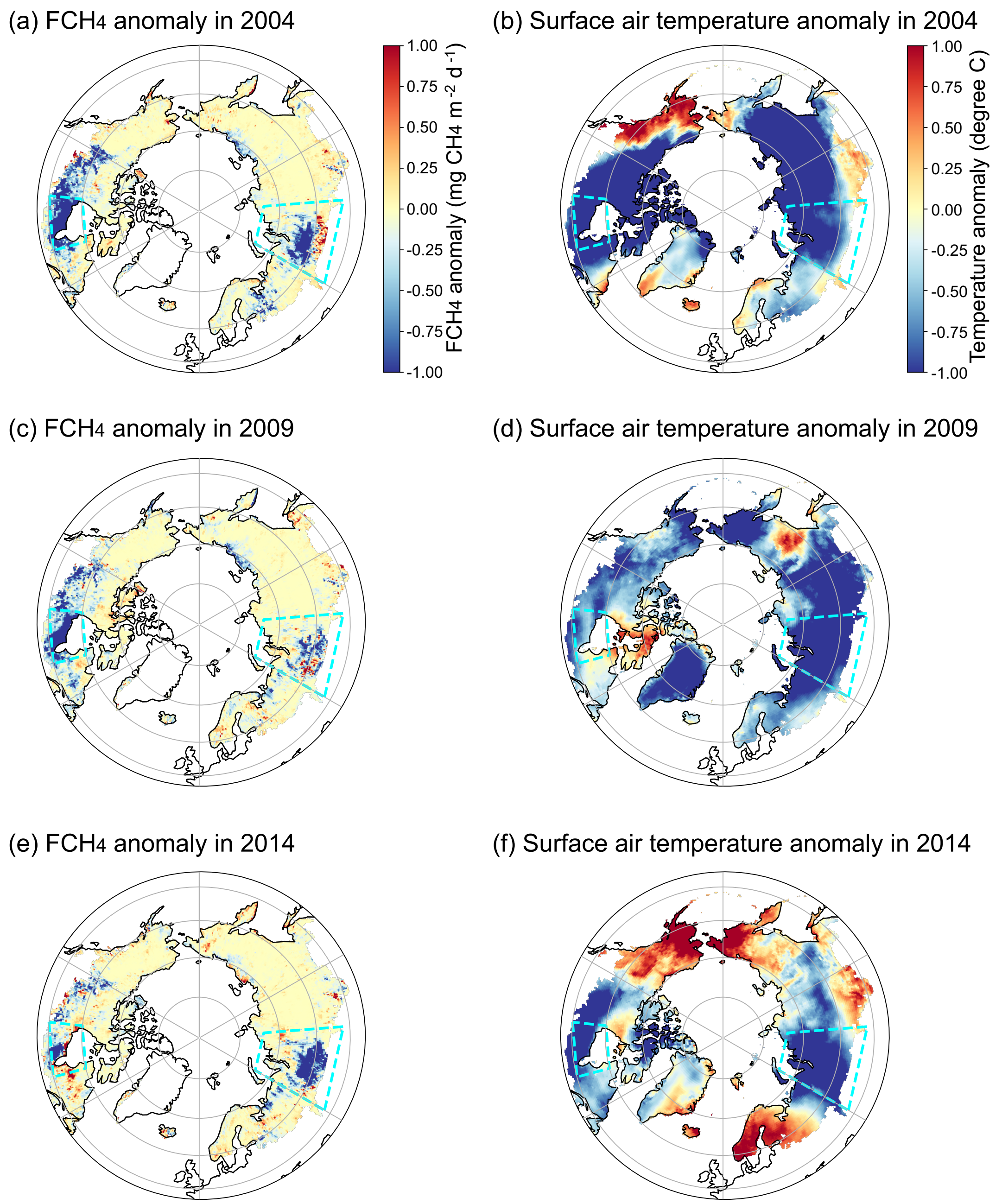

Fig. S7. Wetland methane flux (FCH_4) anomaly, and surface air temperature anomaly in 2004, 2009, and 2014. The anomalies were calculated relative to the multi-year annual mean value from 2002 to 2021. The dashed boxes marked regions in (a)-(f) are two wetland hotpots: Western Siberian lowland and Hudson Bay lowland.

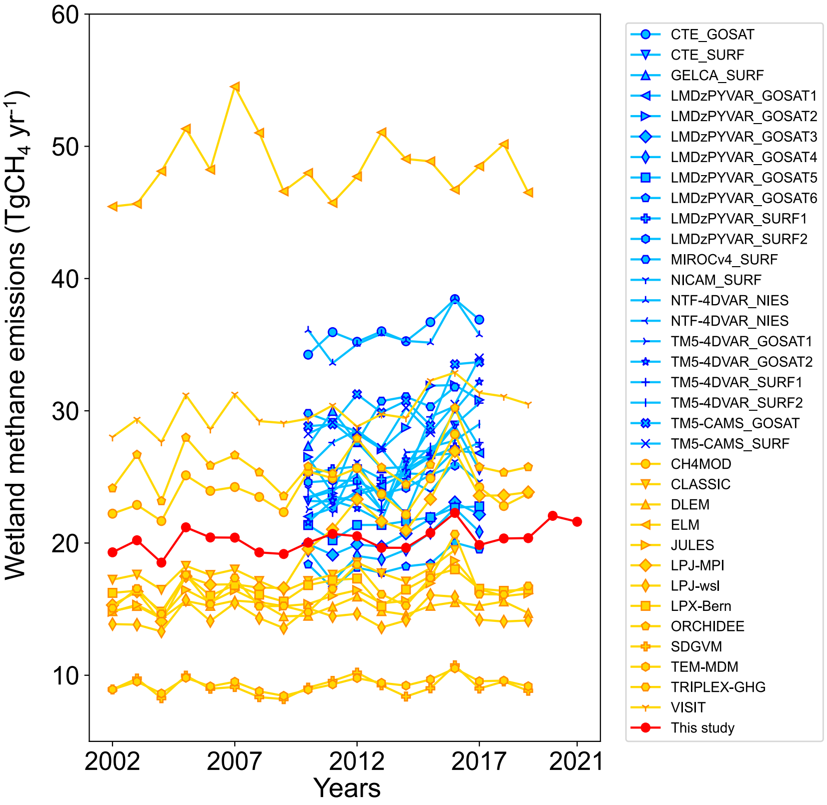

Fig. S8. Inter-annual wetland CH_4 emissions in the Boreal-Arctic estimated by top-down models (blue lines), bottom-up models (gold lines), and this study (the red line).

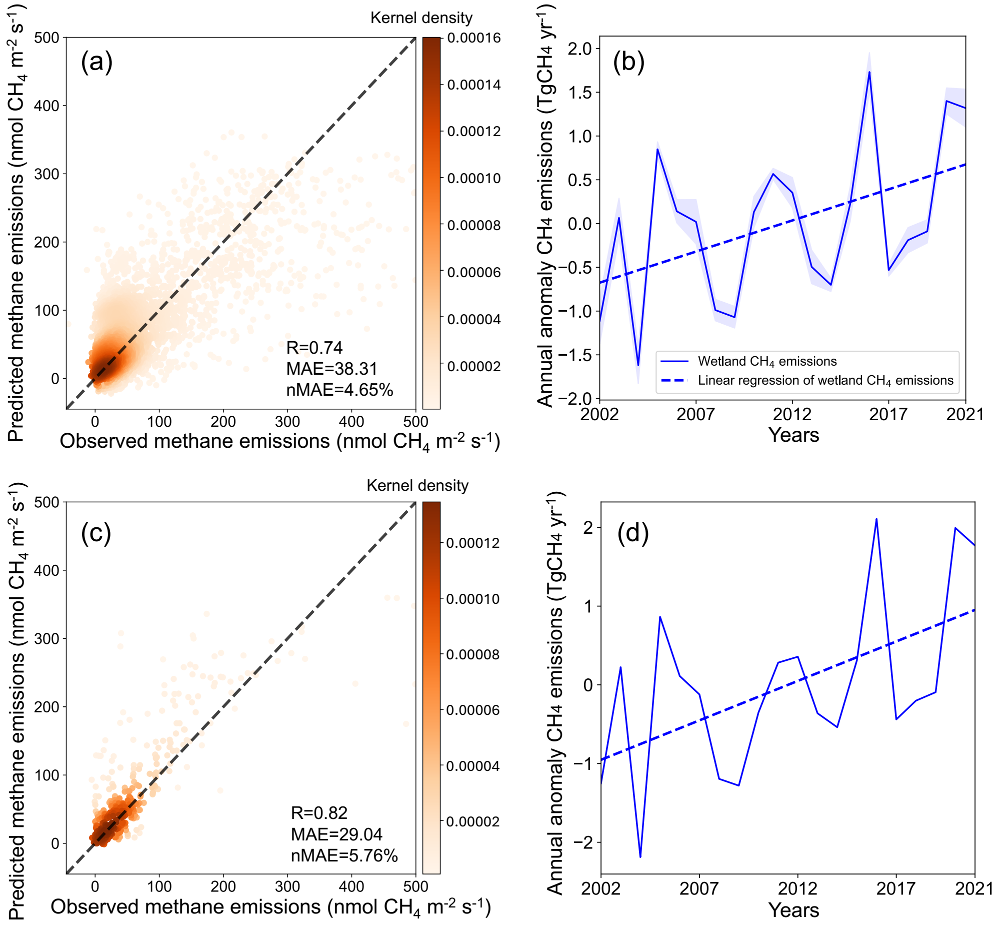

Fig. S9. Model performance and increasing trend of wetland CH_4 emissions in the Boreal-Arctic during 2002-2021 using the leave-one-out validation and the temporal-cross-validation schemes (see Methods). (a) and (c) indicate observed versus model estimated wetland CH_4 emissions using leave-one-out validation scheme and the temporal-cross-validation scheme, respectively. (b) and (d) indicate annual anomaly Boreal-Arctic wetland CH_4 emissions based on leave-one-out validation and the temporal-cross-validation scheme, respectively. The dashed lines in (b) and (d) indicate the linearly regressed trends of wetland CH_4 emissions, and the shaded blue area in (b) indicates the standard deviation in wetland CH_4 dynamics.

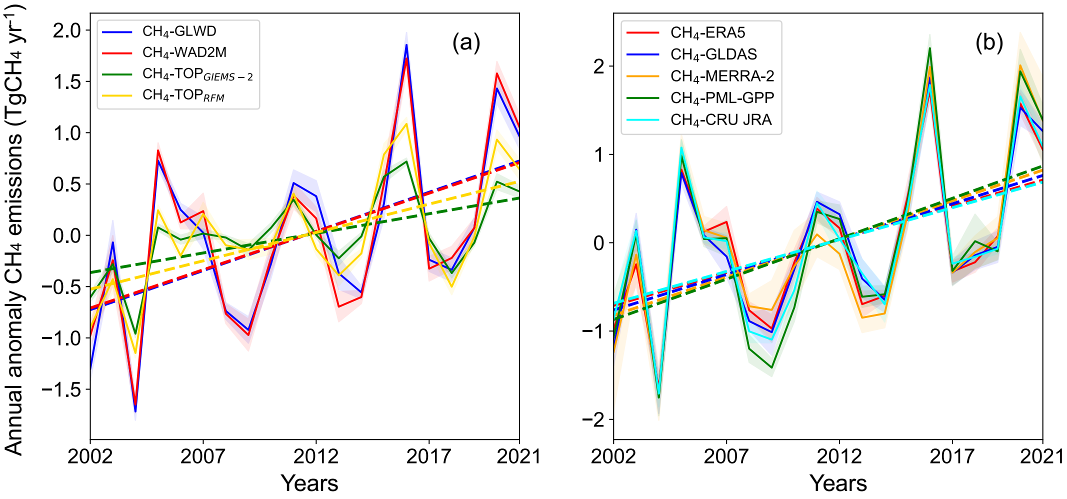

Fig. S10. Wetland CH_4 emissions with increasing trends in the Boreal-Arctic during 2002-2021 using (a) the wetland extent of GLWD, $TOP_{GIEMS-2}$, TOP_{RFW}, and WAD2M, respectively (see Text S3), and (b) the inputting datasets of CRU JRA, GLDAS, MERRA-2, and PML-GPP, respectively (see Text S4). The dashed lines and shaded areas here indicate the linearly regressed trends, and standard deviation in estimated wetland methane dynamics, respectively.

Table S1. Information of eddy covariance sites in this analysis.

Site ID	Wetland type	LAT	LON	Years	Start month	End month	Data DOI	References
CA-SCB	Bog	61.3	-121.3	2014-2017	3	12	DOI: 10.18140/FLX/1669613	[29]
CA-SCC	Bog	61.3	-121.3	2013-2016	3	10	DOI: 10.18140/FLX/1669628	[30]
DE-SfN	Bog	47.8	11.3	2012-2014	1	12	DOI: 10.18140/FLX/1669635	[31]
FI-Si2	Bog	61.8	24.2	2012-2016	1	11	DOI: 10.18140/FLX/1669639	[32]
JP-BBY	Bog	43.3	141.8	2015-2018	2	12	DOI: 10.18140/FLX/1669646	[33]
US-BZB	Bog	64.7	-148.3	2014-2016	4	11	DOI: 10.18140/FLX/1669668	[34]
US-Uaf	Bog	64.9	-147.9	2011-2018	4	10	DOI: 10.18140/FLX/1669701	[35]
Interior	Bog	64.7	-148.3	2013	6	8	DOI: 10.1002/2014JG002683	[36]
Scotty	Bog	61.4	-121.3	2014-2016	6	8	DOI: 10.1111/gcb.13520	[37]
DE-Hte	Fen	54.2	12.2	2011-2018	1	12	DOI: 10.18140/FLX/1669634	[38]
DE-Zrk	Fen	53.9	12.9	2013-2018	1	12	DOI: 10.18140/FLX/1669636	[39]
FI-Lom	Fen	68.0	24.2	2006-2010	1	12	DOI: 10.18140/FLX/1669638	[40]
FI-Sii	Fen	61.8	24.2	2013-2018	1	12	DOI: 10.18140/FLX/1669640	[41]
SE-Deg	Fen	64.2	19.6	2014-2018	1	12	DOI: 10.18140/FLX/1669659	[42]
SE-St1	Fen	68.4	19.1	2012-2014	1	12	DOI: 10.18140/FLX/1669660	[43]
US-BZF	Fen	64.7	-148.3	2014-2016	4	10	DOI: 10.18140/FLX/1669669	[44]
US-Los	Fen	46.1	-90.0	2014-2018	1	12	DOI: 10.18140/FLX/1669682	[45]
Stordalen	Fen	68.3	19.1	2012	6	8	DOI: 10.5194/bg-14-5189-2017	[46]
Churchill	Fen	58.7	-93.8	2009-2012	6	7	DOI: 10.5194/bg-10-4465-2013	[47]
US-DPW	Marsh	28.1	-81.4	2013-2017	1	12	DOI: 10.18140/FLX/1669672	[48]
US-LA2	Marsh	29.9	-90.3	2011-2013	1	12	DOI: 10.18140/FLX/1669681	[49]
US-Myb	Marsh	38.0	-121.8	2011-2018	1	12	DOI: 10.18140/FLX/1669685	[50]
US-ORv	Marsh	40.0	-83.0	2011-2015	1	12	DOI: 10.18140/FLX/1669689	[51]
US-Sne	Marsh	38.0	-121.8	2016-2018	1	12	DOI: 10.18140/FLX/1669693	[52]
US-Tw1	Marsh	38.1	-121.6	2011-2018	1	12	DOI: 10.18140/FLX/1669696	[53]
US-Tw4	Marsh	38.1	-121.6	2013-2018	1	12	DOI: 10.18140/FLX/1669698	[54]
US-WPT	Marsh	41.5	-83.0	2011-2013	1	12	DOI: 10.18140/FLX/1669702	[55]
Stordalen	Marsh	68.4	19.1	2006-2007	7	8	DOI: 10.1029/2008JG000913	[56]
RU-Ch2	Wet tundra	68.6	161.4	2014-2016	1	12	DOI: 10.18140/FLX/1669654	[57,58]
RU-Sam	Wet tundra	72.4	126.5	2011-2014	4	9	DOI:10.18140/FLX/1440185	[59]

US-Beo	Wet tundra	71.3	-156.6	2013-2014	1	12	DOI: 10.18140/FLX/1669664	[60]
US-Bes	Wet tundra	71.3	-156.6	2013-2015	1	12	DOI: 10.18140/FLX/1669665	[61]
US-ICs	Wet tundra	68.6	-149.3	2014-2016	5	10	DOI: 10.18140/FLX/1669678	[62]
US-Ivo	Wet tundra	68.5	-155.8	2013-2016	1	12	DOI: 10.18140/FLX/1669679	[63]
US-NGB	Wet tundra	71.3	-156.6	2012-2018	4	10	DOI: 10.18140/FLX/1669687	[64]
Denali	Wet tundra	63.9	-149.2	2016-2017	5	10	DOI: 10.1029/2018JG004444	[65]

The observations of Interior, Scotty, Stordalen, Churchill, and Denali are obtained from BAWLD-CH_4 dataset [66], and the rest sites are obtained from Fluxnet-CH_4 dataset[67,68].

Table S2. Information of chamber sites used to constrain the model in this study. The datasets are obtained from BAWLD-CH_4 dataset [66] and the datasets in Bao et al.[69]

Site_ID	Wetland type	Lats	Lons	Years	Start month	End month	DOI	References
Athabasca	Bog	55.0	-112.5	2007	5	9	DOI: 10.1111/j.1365-2486.2009.02083.x	[70]
Atqasuk	Marsh	70.5	-157.4	2014	7	8	DOI: 10.1007/s10021-016-9991-0	[71]
Bac_ST	Fen	68.4	19.1	2003-2006	6	9	DOI: 10.5194/bg-7-95-2010<br>DOI: 10.1029/2008JG000703	[72,73]
Barkchar	Bog	57.0	82.6	2013-2014	7	7	DOI: 10.3103/S1068373917050077	[74]
Barkchar	Fen	57.0	82.6	2013-2014	7	7	DOI: 10.3103/S1068373917050077	[74]
Barrow	WetTundra	71.3	-156.6	2007	7	8	DOI: 10.1029/2009JG001283	[75]
Barrow	WetTundra	71	-157	2005	6	8	DOI: 10.1029/2006JG000314	[76]
Bonanza	Fen	65.4	-148.5	2005	7	10	DOI: 10.1029/2007JG000496	[77]
Bonanza	Marsh	64.7	-148.3	2004	5	8	DOI: 10.1029/2005JG000099	[78]
Bonanza	Fen	64.8	-147.9	2005-2013	6	8	DOI: 10.1111/gcb.13612	[79]
Bottemyra	Bog	69.7	29.2	2011	7	7	DOI: 10.3389/fmicb.2015.00356	[80]
Bottemyra	Marsh	69.7	29.2	2011	7	7	DOI: 10.3389/fmicb.2015.00356	[80]
Bro_MB	Bog	45.7	-75.9	2011-2012	6	9	DOI: 10.1002/2013JG002576	[81]
Chokurdakh	WetTundra	70.8	147.4	2004-2006	7	8	DOI: 10.5194/bg-4-985-2007	[82]
Cor_KO	Fen	68.6	161.3	2002	6	9	DOI: 10.1111/j.1365-2486.2005.01023.x	[83]
Des_FE	Fen	62.1	-50.2	2007	6	9	DOI: 10.1111/j.1747-0765.2009.00389.x	[84]
Emm_LH	Fen	81.8	-71.4	2012	6	9	DOI: 10.5194/bg-11-3095-2014	[85]
Fort	Fen	57.0	-111.5	2011-2014	5	10	DOI: 10.1007/s11273-020-09715-2	[86]
Fort	Fen	56.6	-111.3	2015	5	8	DOI: 10.1016/j.scitotenv.2017.01.076	[87]
Fort	Bog	56.4	-111.2	2011-2014	5	10	DOI: 10.1007/s11273-020-09715-2	[86]

Fort	Fen	56.4	-111.2	2011-2015	2011:6<br>2012-2015:5	2011:8<br>2012-2015:10	DOI: 10.1016/j.scitotenv.2017.01.076<br>DOI: 10.1007/s11273-020-09715-2	[86,87]
Hudson	Bog	52.7	-84.0	2013-2014	6	8	-	Harris L.I. unpublished
Hudson	Fen	52.7	-84.0	2013-2014	6	8	-	Harris L.I. unpublished
Innoko	Fen	63.6	-157.7	2011	7	7	DOI: 10.1088/1748-9326/9/10/109601	[88]
Innoko	Bog	63.6	-157.7	2011	7	7	DOI: 10.1088/1748-9326/9/10/109601	[88]
James	Fen	54.1	-72.5	2009-2010	7	8	DOI: 10.1007/s10533-012-9767-3	[89]
James	Bog	53.7	-78.2	2012	7	8	DOI: 10.1016/j.atmosenv.2013.09.044	[90]
Komi	Fen	67.1	63.0	2007-2008	6	8	DOI: 10.5194/bgd-12-13931-2015	[91]
La	Fen	53.6	-77.7	2003	6	8	DOI: 10.1029/2006JG000216	[92]
La	Marsh	53.6	-77.7	2003	6	8	DOI: 10.1029/2006JG000216	[92]
La	Bog	53.6	-77.7	2003	6	8	DOI: 10.1029/2006JG000216	[92]
Lakkasuo	Bog	61.8	24.3	2001	6	9	DOI: 10.5194/bg-17-727-2020	[93]
LekVorkutaKomi	Marsh	67.4	63.4	2001	6	8	DOI: 10.1029/2003GB002054	[94]
Lutose	Bog	59.5	-117.2	2017-2019	5	10	-	Heffernan et al. in prep
Mer_SA	Fen	70	161.6	2005	6	9	DOI: 10.1111/j.1365-2486.2009.01962.x	[95]
Par_WE	Fen	70.8	147.5	2007-2009	6	9	DOI: 10.1029/2010JG001637	[96]
Par_MI	Fen	70.8	147.5	2007-2009	6	9	DOI: 10.1029/2010JG001637	[96]
Par_DR	Fen	70.8	147.5	2007-2009	6	9	DOI: 10.1029/2010JG001637	[96]
Sac_DW	Fen	72.4	126.5	2006	6	9	DOI: 10.1111/j.1365-2486.2010.02232.x	[97]
Sac_LR	Fen	72.4	126.5	2006	6	9	DOI: 10.1029/2007JG000505	[98]
Mye_BB	Bog	65.1	-148.6	2004	6	9	DOI: 10.1029/2007JG000423	[99]
Mye_FF	Fen	65.1	-148.6	2004	6	9	DOI: 10.1029/2007JG000423	[99]
Neleger	WetTundra	62	130	2007	6	8	DOI: 10.1111/j.1747-0765.2009.00389.x	[84]
Northern	Bog	69.5	27.2	2008	6	8	DOI: 10.1111/gcb.12975	[100]
Northern	Fen	68.0	24.2	2008-2010	6	8	https://helda.helsinki.fi/bitstream/handle/10138/228286/ber20-4-489.pdf?sequence=1	[101]
Northern	Fen	67.4	26.7	2012	7	8	DOI: 10.5194/bg-14-799-2017	[102]
Noyabr'sk	Bog	63.2	74.8	2010	7	8	DOI: 10.3103/S0147687412010061	[103]
Pel_G1	Fen	53.9	-78.8	2003	6	9	DOI: 10.1029/2006JG000216	[92]
Pel_G2	Bog	53.6	-77.7	2003	6	9	DOI: 10.1029/2006JG000216	[92]

Pel_G3	Marsh	53.6	-76.1	2003	6	9	DOI: 10.1029/2006JG000216	92
Pir_ZA	Fen	74.5	-21.0	2010	6	9	DOI: 10.1007/s13280-016-0893-3	104
Rhe_BE	Fen	71	-157	2005	6	9	DOI: 10.1029/2006JG000314	76
Scn_ZA	Fen	74.5	-20.6	2005	6	9	DOI: 10.1016/j.soilbio.2011.09.005	105
Siikaneva	Bog	61.8	24.2	2012-2014	6	8	DOI: 10.5194/bg-15-1749-2018	106
Southern	Marsh	61.8	24.3	2008-2010	6	8	https://helda.helsinki.fi/bitstream/handle/10138/228286/ber20-4-489.pdf?sequence=1	101
SouthernFin	Marsh	62.2	23.4	2008-2010	6	8	https://helda.helsinki.fi/bitstream/handle/10138/228286/ber20-4-489.pdf?sequence=1	101
Stordalen	Bog	68.4	19.1	2000, 2003-2006	7,7	7,8	DOI: 10.1029/2001JD001030 DOI: 10.5194/bg-7-95-2010	72,107
Stordalen	Bog	68.4	19.0	2012-2014	7	8	DOI: 10.5194/bg-12-3119-2015	108
Stordalen	Fen	68.4	19.1	2000, 2003-2006	2000:7, 2003-2006:7	2000:7, 2003-2006:8	DOI: 10.1029/2001JD001030 DOI: 10.1016/j.soilbio.2007.01.019 DOI: 10.5194/bg-7-95-2010	109
Stordalen	Marsh	68.4	19.0	2012-2015	7	8	DOI: 10.5194/bg-12-3119-2015	108
Str_SC	Fen	46.7	-71.2	2004	6	9	DOI: 10.1007/s10021-005-0070-1	110
Str_ZA	Fen	74.5	-20.5	2011-2013	6	9	DOI: 10.1016/j.soilbio.2011.09.005	105
Tag_ZA	Fen	74.5	-20.6	2007-2009	6	9	DOI: 10.3402/tellusb.v65i0.19722	111
Tak_WG	Marsh	62.3	129.5	2005	6	9	DOI: 10.1029/2007JG000521	112
Tanana	Bog	64.6	-148.3	2004	7	8	DOI: 10.1029/2007JG000423	99
Taz	Bog	67.4	78.9	2010	7	8	DOI: 10.17816/edgcc211-16	113
Teslin	Bog	60.1	-131.4	2013	7	8	DOI: 10.1038/nclimate3328	114
Tur_AP	Fen	65.4	-148.5	2005-2006	6	9	DOI: 10.1029/2007JG000496	77
Van_SM	Marsh	62.4	130.0	2005	6	9	DOI: 10.1016/j.agrformet.2008.08.008	115
Van_SR	Fen	70.8	147.4	2004-2006	6	9	DOI: 10.5194/bg-4-985-2007 DOI: 10.1029/2005JG000010	82,116
Van_ST	Fen	62.4	130.0	2005	6	9	DOI: 10.1016/j.agrformet.2008.08.008	115
Wandering	Bog	55.4	-112.5	2011-2013	5	9	DOI: 10.1007/s10021-014-9795-z	117
WesternNew	Bog	48.3	-58.7	2013	7	8	DOI: 10.1088/1748-9326/9/10/105005	118
WesternSib	Fen	56.2	78.4	2013	7	8	DOI: 10.1134/S1062359016020060	119

WesternSib	Marsh	56.2	78.4	2013	7	8	DOI: 10.1134/S1062359016020060	[119]
WesternSib	Fen	55.2	78.2	2013	7	8	DOI: 10.1134/S1062359016020060	[119]
WesternSib	Fen	56.5	78.3	2011	7	8	DOI: 10.1088/1748-9326/9/4/045008	[120]
WesternSib	Fen	56.4	78.8	2011	7	8	DOI: 10.1088/1748-9326/9/4/045008	[120]
WesternSib	Fen	55.8	78.4	2013	7	8	DOI: 10.1134/S1062359016020060	[119]
Wil_SI	Fen	72.4	126.5	2003-2004	6	9	DOI: 10.1111/j.1365-2486.2008.01586.x	[121]
Yakutsk	Fen	62.3	129.6	2004-2006	7	8	DOI: 10.1016/j.agrformet.2008.08.008	[115]
Yakutsk	Marsh	62.2	129.6	2004-2006	7	8	DOI: 10.1016/j.agrformet.2008.08.008	[115]
Yellowknife	Bog	62.5	-114.5	2014	7	7	DOI: 10.1038/nclimate3328	[114]
Zon_BE	Fen	71.5	-157.0	2007	6	9	DOI: 10.1029/2009GB003487	[122]

Table S3. Site id, name, and location of the atmospheric methane content observation sites, and the mean values and trends of methane contents. ($^{*}p$<0.1, $^{**}p$<0.05, and $^{***}p$<0.01).

**Site_id**	**Site_name**	**LAT**	**LON**	**Mean CH4 content (ppb)**	**trend**
ALT	Alert, Nunavut, Canada	82.45	-62.51	1898.467	0.543***
BAL	Baltic Sea, Poland	55.35	17.22	1895.737	0.259***
BRW	Barrow Atmospheric Baseline Observatory, United States	71.32	-156.61	1909.031	0.544***
CBA	Cold Bay, Alaska, United States	55.21	-162.72	1897.286	0.612***
HPB	Hohenpeissenberg, Germany	47.8	11.02	1946.113	0.624***
HUN	Hegyhatsal, Hungary	46.95	16.65	1945.406	0.476***
ICE	Storhofdi, Vestmannaeyjar, Iceland	63.4	-20.29	1892.464	0.540***
KZD	Sary Taukum, Kazakhstan	44.08	76.87	1880.285	0.395**
KZM	Plateau Assy, Kazakhstan	43.25	77.99	1846.999	0.137**
LLB	Lac La Biche, Alberta, Canada	54.95	112.47	1941.489	0.820
MHD	Mace Head, County Galway, Ireland	53.33	-9.9	1887.412	0.553***
OXK	Ochsenkopf, Germany	50.03	11.81	1929.801	0.530***
PAL	Pallas-Sammaltunturi, GAW Station, Finland	67.97	24.12	1911.230	0.528***
SHM	Shemya Island, Alaska, United States	52.71	174.13	1891.924	0.556***
SUM	Summit, Greenland	72.6	38.42	1890.394	0.567***
TIK	Hydrometeorological Observatory of Tiksi, Russia	71.6	128.89	1938.278	0.774***
UUM	Ulaan Uul, Mongolia	44.45	111.1	1891.359	0.509***

ZEP	Ny-Alesund, Svalbard, Norway and Sweden	78.91	11.89	1899.470	0.527***

The observations are obtained from: Lan, X., E.J. Dlugokencky, J.W. Mund, A.M. Crotwell, M.J. Crotwell, E. Moglia, M. Madronich, D. Neff and K.W. Thoning (2022), Atmospheric Methane Dry Air Mole Fractions from the NOAA GML Carbon Cycle Cooperative Global Air Sampling Network, 1983-2021, Version: 2022-07-28, https://doi.org/10.15138/VNCZ-M766.

Table S4. Sites that cover the year 2016 and its adjacent years, and show anomaly high temperature in 2016.

**Site name**	**Years**	**TA mean (K)**	**TA anomaly in 2016 (K)**	**FCH_4 mean (nmol CH_4 m^{-2} s^{-1})**	**FCH_4 anomaly in 2016 (nmol CH_4 m^{-2} s^{-1})**
CASCB	2014-2017	272.70	0.16	36.28	0.67
CASCC	2013-2016	272.70	0.16	30.90	3.06
USBZB	2014-2016	272.36	0.77	46.25	6.41
USUAF	2011-2018	271.74	1.87	2.71	2.14
DEHTE	2011-2018	282.98	0.14	158.98	16.91
USBZF	2014-2016	272.36	0.77	48.44	16.56
USLOS	2014-2018	277.71	1.22	18.85	5.84
RUCH2	2014-2016	263.71	1.18	25.67	6.35
USICS	2014-2016	264.84	0.72	11.09	-0.86
USIVO	2013-2016	264.64	1.21	15.18	2.58
USNGB	2012-2018	265.25	1.53	11.17	1.44

Table S5. Annual wetland methane emissions and trends (with *p* values obtained from two-sided *t*-test) estimated by different models in the Boreal-Arctic region ($^{*}p<0.1$, $^{**}p<0.05$, and $^{***}p<0.01$).

**Model type**	**FCH_4 data source**	**Trend (Tg CH_4 yr^{-2})**		**Annual emission (Tg CH_4 yr^{-2})**
		Boreal arctic	*p* value	Boreal arctic
**Top-down models**	CTE_GOSAT	0.414**	0.019	36.090±1.197
	CTE_SURF	0.619*	0.061	24.666±2.073
	GELCA_SURF	-0.229	0.624	27.503±1.530
	LMDzPYVAR_GOSAT1	0.714***	2.685×10^{-5}	24.646±1.673
	LMDzPYVAR_GOSAT2	0.996***	0.007	28.628±2.675
	LMDzPYVAR_GOSAT3	0.505***	0.004	20.808±1.313
	LMDzPYVAR_GOSAT4	0.520**	0.027	19.755±1.559

	LMDzPYVAR_GOSAT5	0.289***	0.006	21.670±0.767
	LMDzPYVAR_GOSAT6	0.315**	0.037	18.388±0.981
	LMDzPYVAR_SURF1	0.262*	0.087	25.789±0.762
	LMDzPYVAR_SURF2	0.145	0.299	24.698±0.632
	MIROCv4_SURF	0.422	0.108	30.061±1.284
	NICAM_SURF	0.212	0.346	24.415±1.265
	NTF-4DVAR_NIES_GOSAT	0.259	0.248	35.653±1.280
	NTF-4DVAR_NIES_SURF	0.297	0.327	27.630±1.707
	TM5-4DVAR_GOSAT1	1.183***	0.001	25.613±2.957
	TM5-4DVAR_GOSAT2	1.488***	0.001	25.523±2.723
	TM5-4DVAR_SURF1	0.617***	0.008	25.138±1.675
	TM5-4DVAR_SURF2	0.802***	0.005	25.592±2.120
	TM5-CAMS_GOSAT	0.601**	0.035	30.721±1.857
	TM5-CAMS_SURF	0.591*	0.059	29.513±1.969
**Bottom-up models**	CH4MOD	0.100	0.178	23.977±1.554
	CLASSIC	-0.774***	0.007	14.682±6.604
	DLEM	0.009	0.693	15.126±0.470
	ELM	0.009	0.938	48.510±2.323
	JULES	0.099**	0.016	15.940±0.919
	LPJ-MPI	0.635***	1.370×10^{-7}	19.885±3.612
	LPJ-wsl	0.031	0.428	14.474±0.812
	LPX-Bern	0.045	0.238	16.526±0.801
	ORCHIDEE	0.067	0.396	25.858±1.624
	SDGVM	0.020	0.543	9.175±0.674
	TEM-MDM	0.037	0.106	9.330±0.481
	TRIPLEX	0.097	0.161	16.400±1.461
	VISIT	0.164***	0.008	30.017±1.403
	**This study**	0.086**	0.017	20.316±0.938

Text S1. Combination of the BAWLD and WAD2M datasets

The percentage of each wetland type within each upscaled wetland grid cell was determined by combining BAWLD and WAD2M datasets. The WAD2M dataset provided the temporal dynamics of wetland extent information but without differentiating wetland types[1], while the BAWLD dataset recorded the wetland extent for each wetland type but was temporally static[2]. Here we used the wetland type-specific extent information in BAWLD to calculate the fraction of each wetland type relative to its total wetland area. Then we derived the temporally varied wetland extent for each wetland type by multiplying the wetland extent of WAD2M by the wetland type-specific fraction calculated from the first step (Eq. 1). By doing so, we assume that the fraction of each wetland type relative to its total wetland area does not change over time because we do not have information regarding temporally varying wetland types. For the upscaling, we considered all wetland grid cells in the BAWLD dataset that provided wetland type information.

$$E_{i,t}(s) = E_{WAD2M,t}(s) \times \frac{E_{BAWLD,i}(s)}{\sum_{j=1}^{n} E_{BAWLD,j}(s)} \quad (1)$$

where $E_{i,t}(s)$ is the wetland extent of the i-th wetland type used for upscaling CH_4 in the s-th grid cell at time t; $E_{WAD2M,t}(s)$ is the wetland extent for the WAD2M dataset at time t; $E_{BAWLD,i}(s)$ and $E_{BAWLD,j}(s)$ are the wetland extent of the i-th and j-th wetland types in the BAWLD dataset, respectively, and n is the total number of wetland types within the s-th grid cell of the BAWLD dataset.

Text S2. Inferring causal relationships

In the Causal-ML approach, we first used a data-driven causality inference method to identify the causal relationships between CH_4 emission and its abiotic and biotic drivers[3,4]. A causal relationship is present between two variables if changes in one variable (e.g., temperature) directly result in changes in another variable (e.g., wetland CH_4 emissions)[4-7], all else being equal. The literature describes three ways to infer causal relationships: interventional experiments[8], process-oriented model simulations[9,10], and data-driven causality inference[4,11,12]. Interventional experiments intervene on the variable of interest while simultaneously maintaining all other factors equal, and then tests the impact of this intervention on the target variable[8]. Interventional experiments across large spatial scale remain challenging[13]. Process-oriented model simulations (i.e., computer simulations) control confounding factors and focus on the causal relationship of interest, but the model itself requires substantial expert knowledge and reasonable model structure and parameterization. Unfortunately, the current generation of bottom-up biogeochemical models are highly uncertain and poorly constrained with observed wetland CH_4 emissions (see the main text). Therefore, a data-driven causality inference method was selected for analyzing causal processes in wetland CH_4 emissions.

Specifically, we used the PCMCI method to identify the causal relationships of wetland CH_4 emissions. Wetland CH_4 emissions are controlled by multiple abiotic and biotic factors, and these processes can be asynchronous[3,14-16]. The PCMCI method is

particularly suitable for inferring such multi-variate controlled and time-lagged causal relationships[4,6,17,18], and has been used for understanding temporal dynamics of Earth system processes beyond wetland CH_4 emissions[3], such as climate systems[4,11,18,19] and land-atmosphere interactions[12,20]. The method contains two steps, PC (named after Peter Spirtes and Clark Glymour[21]) and MCI (i.e., momentary conditional independence[18]). The first step, PC, iteratively identifies a smaller size of relevant necessary confounders that affected the inferred causal relationship between two variables of interest (e.g., temperature and CH_4 flux) through conditional independence tests. Subsequently, the momentary conditional independence tests are used to detect and quantify the causal strength between the two variables of interest by removing the confounding effects from the identified necessary confounders in the PC step[17,18]. By avoiding conditioning on high-dimensional variables, the PCMCI improves the detection power of causal relationships[17,18]. We considered abiotic and biotic variables (see the input datasets in the main text) that have reliable global observations or estimates and have been suggested to be mechanistically related to wetland CH_4 emissions by previous studies[14,22]. The high predictability of the Causal-ML demonstrated that the input variables we selected explained the majority of the variance in wetland CH_4 dynamics. While we acknowledge that factors beyond the ones considered here could also affect wetland CH_4 emissions (e.g., oxygen availability, soil pH, and ferric iron and sulfate reducers[22]), the paucity of observations from site to global scale impedes us from including those additional variables for the causality inference and upscaling.

Text S3. Sensitivity of CH_4 dynamics to wetland extent datasets

We used three additional wetland datasets to discuss the sensitivity of wetland CH_4 dynamics to uncertain wetland extent, including the static wetland dataset derived from Global Lakes and Wetlands Database (GLWD)[23], and temporally dynamic wetlands[24] estimated by the TOPography-based hydrological MODEL (TOPMODEL). The GLWD data was derived by merging multiple surveys, maps, and inventories of lake and reservoirs, small water bodies, and wetlands. We used GLWD after excluding lakes, rivers, and reservoirs. In addition to GLWD, we used the temporally varied wetland extent derived from the TOPMODEL model[24]. The TOPMODEL model treated continuous or regularly saturated grid-cells as wetlands. By assuming that water pathways were mainly determined by topography, the model used a compound topographic index to estimate the water table depth and thus the wetland fraction in each grid-cell[24,25]. The modeled wetland extent was temporally varied with a monthly resolution, and was calibrated by observation-based data of Global Inundation Extent from Multi-Satellites (GIEMS-2) and Regularly Flooded Wetland (RFW)[26], respectively. The two modeled wetland datasets constrained by different observations were referred to as $TOP_{GIEMS\text{-}2}$ and TOP_{RFW}, respectively. We did not directly use the GIEMS-2 data due to its limited temporal coverage[27]. To focus on the sensitivity of wetland CH_4 emission dynamics to wetland extent changes, we kept the CH_4 emission intensity the same as in our baseline analysis, while replacing WAD2M with GLWD,

$TOP_{GIEMS-2}$, and TOP_{RFW}, respectively. The upscaled wetland CH_4 emissions using different wetland extent datasets during 2002-2021 were compared in Fig. S12.

Text S4. Sensitivity of CH_4 dynamics to inputting datasets

We conducted four groups of sensitivity experiments to confirm that the increasing trend and strong interannual variations of wetland CH_4 emissions were robust given uncertainty in the input datasets. Specifically, we first replaced the originally-used input variables of air temperature, air pressure, precipitation, and wind speed with those of the CRU JRA dataset, while keeping the other factors including soil conditions, GPP, snow, and wetlands the same. Then we upscaled the wetland CH_4 emissions during 2002-2021 using the updated dataset and compared the regional trend and interannual variations of wetland CH_4 emissions with those in the main text. Similarly, we also used the datasets from GLDAS and MERRA-2 which further provided soil temperature and soil wetness for wetland CH_4 upscaling. Additionally, we replaced the GPP used in the main text with that of PML-GPP, which was derived from an empirical process-model that considered the CO_2 fertilization effects, and was constrained by multi-source remote sensing observations[28]. The upscaled wetland CH_4 emissions using different sources of input variables during 2002-2021 were compared in Fig. S13, and had consistent increasing trends in wetland CH_4 emissions.

References:

1 Zhang, Z. *et al.* Development of the global dataset of Wetland Area and Dynamics for Methane Modeling (WAD2M). *Earth System Science Data* **13**, 2001-2023 (2021).

2 Olefeldt, D. *et al.* The Boreal–Arctic Wetland and Lake Dataset (BAWLD). *Earth system science data* **13**, 5127-5149 (2021).

3 Yuan, K. *et al.* Causality guided machine learning model on wetland CH4 emissions across global wetlands. *Agricultural and Forest Meteorology* **324**, 109115 (2022).

4 Runge, J. *et al.* Inferring causation from time series in Earth system sciences. *Nature communications* **10**, 1-13 (2019).

5 Pearl, J., Glymour, M. & Jewell, N. P. *Causal inference in statistics: A primer*. (John Wiley & Sons, 2016).

6 Runge, J., Gerhardus, A., Varando, G., Eyring, V. & Camps-Valls, G. Causal inference for time series. *Nature Reviews Earth & Environment*, 1-19 (2023).

7 Eichler, M. Causal inference in time series analysis. *Causality: Statistical perspectives and applications*, 327-354 (2012).

8 Imbens, G. W. & Rubin, D. B. *Causal inference in statistics, social, and biomedical sciences*. (Cambridge University Press, 2015).

9 Li, F. *et al.* Vegetation clumping modulates global photosynthesis through adjusting canopy light environment. *Global Change Biology* **29**, 731-746 (2023).

10 Gillett, N. P. *et al.* The detection and attribution model intercomparison project (DAMIP v1. 0) contribution to CMIP6. *Geoscientific Model Development* **9**, 3685-3697 (2016).

11 Li, F. *et al.* Wetter California projected by CMIP6 models with observational constraints under a high GHG emission scenario. *Earth's Future* **10**, e2022EF002694 (2022).

12 Yuan, K., Zhu, Q., Riley, W. J., Li, F. & Wu, H. Understanding and reducing the uncertainties of land surface energy flux partitioning within CMIP6 land models. *Agricultural and Forest Meteorology* **319**, 108920 (2022).

13 Runge, J. *et al.* Inferring causation from time series in Earth system sciences. *Nature communications* **10**, 2553 (2019).

14 Knox, S. H. *et al.* Identifying dominant environmental predictors of freshwater wetland methane fluxes across diurnal to seasonal time scales. *Global change biology* **27**, 3582-3604 (2021).

15 Sturtevant, C. *et al.* Identifying scale-emergent, nonlinear, asynchronous processes of wetland methane exchange. *Journal of Geophysical Research: Biogeosciences* **121**, 188-204 (2016).

16 Chang, K.-Y. *et al.* Substantial hysteresis in emergent temperature sensitivity of global wetland CH4 emissions. *Nature communications* **12**, 1-10 (2021).

17 Runge, J., Nowack, P., Kretschmer, M., Flaxman, S. & Sejdinovic, D. Detecting and quantifying causal associations in large nonlinear time series datasets. *Science advances* **5**, eaau4996 (2019).

18 Runge, J. *et al.* Identifying causal gateways and mediators in complex spatio-temporal systems. *Nature communications* **6**, 8502 (2015).

19 Kretschmer, M., Coumou, D., Donges, J. F. & Runge, J. Using causal effect networks to analyze different Arctic drivers of midlatitude winter circulation. *Journal of climate* **29**, 4069-4081 (2016).

20 Krich, C. *et al.* Estimating causal networks in biosphere–atmosphere interaction with the PCMCI approach. *Biogeosciences* **17**, 1033-1061 (2020).

21 Spirtes, P., Glymour, C. N. & Scheines, R. *Causation, prediction, and search*. (MIT press,

2000).
22 Le Mer, J. & Roger, P. Production, oxidation, emission and consumption of methane by soils: a review. *European journal of soil biology* **37**, 25-50 (2001).
23 Lehner, B. & Döll, P. Development and validation of a global database of lakes, reservoirs and wetlands. *Journal of hydrology* **296**, 1-22 (2004).
24 Xi, Y. *et al.* Gridded maps of wetlands dynamics over mid-low latitudes for 1980–2020 based on TOPMODEL. *Scientific Data* **9**, 347 (2022).
25 Xi, Y., Peng, S., Ciais, P. & Chen, Y. Future impacts of climate change on inland Ramsar wetlands. *Nature Climate Change* **11**, 45-51 (2021).
26 Tootchi, A., Jost, A. & Ducharne, A. Multi-source global wetland maps combining surface water imagery and groundwater constraints. *Earth System Science Data* **11**, 189-220 (2019).
27 Prigent, C., Jimenez, C. & Bousquet, P. Satellite-derived global surface water extent and dynamics over the last 25 years (GIEMS-2). *Journal of Geophysical Research: Atmospheres* **125**, e2019JD030711 (2020).
28 Zhang, Y. *et al.* Coupled estimation of 500 m and 8-day resolution global evapotranspiration and gross primary production in 2002–2017. *Remote sensing of environment* **222**, 165-182 (2019).
29 Sonnentag, O. & Helbig, M. *FLUXNET-CH4 CA-SCB Scotty Creek Bog*, 2020).
30 Sonnentag, O. & Helbig, M. *FLUXNET-CH4 CA-SCC Scotty Creek Landscape*, <https://doi:10.18140/FLX/1669628> (2020).
31 Schmid, H. P. & Klatt, J. *FLUXNET-CH4 DE-SfN Schechenfilz Nord*, 2020).
32 Vesala, T., Tuittila, E.-S., Mammarella, I. & Alekseychik, P. *FLUXNET-CH4 FI-Si2 Siikaneva-2 Bog*, 2020).
33 Ueyama, M., Hirano, T. & Kominami, Y. *FLUXNET-CH4 JP-BBY Bibai bog*, 2020).
34 Euskirchen, E. & Edgar, C. *FLUXNET-CH4 US-BZB Bonanza Creek Thermokarst Bog*, 2020).
35 Iwata, H., Ueyama, M. & Harazono, Y. *FLUXNET-CH4 US-Uaf University of Alaska, Fairbanks*, 2020).
36 Euskirchen, E. S., Edgar, C., Turetsky, M., Waldrop, M. P. & Harden, J. W. Differential response of carbon fluxes to climate in three peatland ecosystems that vary in the presence and stability of permafrost. *Journal of Geophysical Research: Biogeosciences* **119**, 1576-1595 (2014).
37 Helbig, M. *et al.* The positive net radiative greenhouse gas forcing of increasing methane emissions from a thawing boreal forest-wetland landscape. *Global change biology* **23**, 2413-2427 (2017).
38 Koebsch, F. & Jurasinski, G. *FLUXNET-CH4 DE-Hte Huetelmoor*, 2020).
39 Sachs, T. & Wille, C. *FLUXNET-CH4 DE-Zrk Zarnekow*, 2020).
40 Lohila, A. *et al. FLUXNET-CH4 FI-Lom Lompolojankka*, 2020).
41 Vesala, T., Tuittila, E.-S., Mammarella, I. & Rinne, J. *FLUXNET-CH4 FI-Sii Siikaneva*, 2020).
42 Nilsson, M. B. & Peichl, M. *FLUXNET-CH4 SE-Deg Degero*, 2020).
43 Jansen, J., Friborg, T., Jammet, M. & Crill, P. *FLUXNET-CH4 SE-St1 Stordalen grassland*, 2020).
44 Euskirchen, E. & Edgar, C. *FLUXNET-CH4 US-BZF Bonanza Creek Rich Fen*, 2020).
45 Desai, A. R. & Thom, J. *FLUXNET-CH4 US-Los Lost Creek*, 2020).
46 Jammet, M. *et al.* Year-round CH 4 and CO 2 flux dynamics in two contrasting freshwater

ecosystems of the subarctic. *Biogeosciences* **14**, 5189-5216 (2017).
47 Hanis, K., Tenuta, M., Amiro, B. & Papakyriakou, T. Seasonal dynamics of methane emissions from a subarctic fen in the Hudson Bay Lowlands. *Biogeosciences* **10**, 4465-4479 (2013).
48 Hinkle, C. R. & Bracho, R. *FLUXNET-CH4 US-DPW Disney Wilderness Preserve Wetland*, 2020).
49 Holm, G. O. *et al. FLUXNET-CH4 US-LA2 Salvador WMA Freshwater Marsh*, 2020).
50 Matthes, J. H. *et al. FLUXNET-CH4 US-Myb Mayberry Wetland*, 2020).
51 Bohrer, G. & Morin, T. H. *FLUXNET-CH4 US-ORv Olentangy River Wetland Research Park*, 2020).
52 Shortt, R., Hemes, K., Szutu, D., Verfaillie, J. & Baldocchi, D. *FLUXNET-CH4 US-Sne Sherman Island Restored Wetland*, 2020).
53 Valach, A. C. *et al. FLUXNET-CH4 US-Tw1 Twitchell Wetland West Pond*, 2020).
54 Eichelmann, E. *et al. FLUXNET-CH4 US-Tw4 Twitchell East End Wetland*, 2020).
55 Chen, J. & Chu, H. *FLUXNET-CH4 US-WPT Winous Point North Marsh*, 2020).
56 Jackowicz-Korczyński, M. *et al.* Annual cycle of methane emission from a subarctic peatland. *Journal of Geophysical Research: Biogeosciences* **115** (2010).
57 Göckede, M. *FLUXNET-CH4 RU-Ch2 Chersky reference*, 2020).
58 Göckede, M. *et al.* Negative feedback processes following drainage slow down permafrost degradation. *Global change biology* **25**, 3254-3266 (2019).
59 Lars Kutzbach, T. S., Julia Boike, Christian Wille, Peter Schreiber, Moritz Langer, Eva-Maria Pfeiffer, Benjamin Runkle, Günter Stoof, David Holl, Lars Heling, Norman Rüggen. (2020).
60 Zona, D. & Oechel, W. C. *FLUXNET-CH4 US-Beo Barrow Environmental Observatory (BEO) tower*, 2020).
61 Zona, D. & Oechel, W. C. *FLUXNET-CH4 US-Bes Barrow-Bes (Biocomplexity Experiment South tower)*, 2020).
62 Euskirchen, E., Bret-Harte, M. & Edgar, C. *FLUXNET-CH4 US-ICs Imnavait Creek Watershed Wet Sedge Tundra*, 2020).
63 Zona, D. & Oechel, W. C. *FLUXNET-CH4 US-Ivo Ivotuk*, 2020).
64 Torn, M. & Dengel, S. *FLUXNET-CH4 US-NGB NGEE Arctic Barrow*, 2020).
65 Taylor, M., Celis, G., Ledman, J., Bracho, R. & Schuur, E. Methane efflux measured by eddy covariance in Alaskan upland tundra undergoing permafrost degradation. *Journal of Geophysical Research: Biogeosciences* **123**, 2695-2710 (2018).
66 Kuhn, M. A. *et al.* BAWLD-CH 4: a comprehensive dataset of methane fluxes from boreal and arctic ecosystems. *Earth System Science Data* **13**, 5151-5189 (2021).
67 Delwiche, K. B. *et al.* FLUXNET-CH4: A global, multi-ecosystem dataset and analysis of methane seasonality from freshwater wetlands. *Earth System Science Data Discussions*, 1-111 (2021).
68 Knox, S. H. *et al.* FLUXNET-CH4 synthesis activity: Objectives, observations, and future directions. *Bulletin of the American Meteorological Society* **100**, 2607-2632 (2019).
69 Bao, T., Jia, G. & Xu, X. Wetland Heterogeneity Determines Methane Emissions: A Pan-Arctic Synthesis. *Environmental Science & Technology* **55**, 10152-10163 (2021).
70 Long, K. D., Flanagan, L. B. & Cai, T. Diurnal and seasonal variation in methane emissions in a northern Canadian peatland measured by eddy covariance. *Global change biology* **16**, 2420-

2435 (2010).
71 Davidson, S. J. *et al.* Vegetation type dominates the spatial variability in CH4 emissions across multiple Arctic tundra landscapes. *Ecosystems* **19**, 1116-1132 (2016).
72 Bäckstrand, K. *et al.* Annual carbon gas budget for a subarctic peatland, Northern Sweden. *Biogeosciences* **7**, 95-108 (2010).
73 Bäckstrand, K., Crill, P. M., Mastepanov, M., Christensen, T. R. & Bastviken, D. Total hydrocarbon flux dynamics at a subarctic mire in northern Sweden. *Journal of geophysical research: Biogeosciences* **113** (2008).
74 Veretennikova, E. & Dyukarev, E. Diurnal variations in methane emissions from West Siberia peatlands in summer. *Russian Meteorology and Hydrology* **42**, 319-326 (2017).
75 von Fischer, J. C., Rhew, R. C., Ames, G. M., Fosdick, B. K. & von Fischer, P. E. Vegetation height and other controls of spatial variability in methane emissions from the Arctic coastal tundra at Barrow, Alaska. *Journal of Geophysical Research: Biogeosciences* **115** (2010).
76 Rhew, R. C., Teh, Y. A. & Abel, T. Methyl halide and methane fluxes in the northern Alaskan coastal tundra. *Journal of Geophysical Research: Biogeosciences* **112** (2007).
77 Turetsky, M. *et al.* Short-term response of methane fluxes and methanogen activity to water table and soil warming manipulations in an Alaskan peatland. *Journal of Geophysical Research: Biogeosciences* **113** (2008).
78 Wickland, K. P., Striegl, R. G., Neff, J. C. & Sachs, T. Effects of permafrost melting on CO2 and CH4 exchange of a poorly drained black spruce lowland. *Journal of Geophysical Research: Biogeosciences* **111** (2006).
79 Olefeldt, D. *et al.* A decade of boreal rich fen greenhouse gas fluxes in response to natural and experimental water table variability. *Global change biology* **23**, 2428-2440 (2017).
80 Liebner, S. *et al.* Shifts in methanogenic community composition and methane fluxes along the degradation of discontinuous permafrost. *Frontiers in Microbiology* **6**, 356 (2015).
81 Brown, M. G., Humphreys, E. R., Moore, T. R., Roulet, N. T. & Lafleur, P. M. Evidence for a nonmonotonic relationship between ecosystem-scale peatland methane emissions and water table depth. *Journal of Geophysical Research: Biogeosciences* **119**, 826-835 (2014).
82 Van der Molen, M. *et al.* The growing season greenhouse gas balance of a continental tundra site in the Indigirka lowlands, NE Siberia. *Biogeosciences* **4**, 985-1003 (2007).
83 Corradi, C., Kolle, O., Walter, K., Zimov, S. & Schulze, E. D. Carbon dioxide and methane exchange of a north-east Siberian tussock tundra. *Global Change Biology* **11**, 1910-1925 (2005).
84 Desyatkin, A. R. *et al.* CH4 emission from different stages of thermokarst formation in Central Yakutia, East Siberia. *Soil Science and Plant Nutrition* **55**, 558-570 (2009).
85 Emmerton, C. *et al.* The net exchange of methane with high Arctic landscapes during the summer growing season. *Biogeosciences* **11**, 3095-3106 (2014).
86 Bienida, A. *et al.* Methane emissions from fens in Alberta's boreal region: reference data for functional evaluation of restoration outcomes. *Wetlands Ecology and Management* **28**, 559-575 (2020).
87 Murray, K. R., Barlow, N. & Strack, M. Methane emissions dynamics from a constructed fen and reference sites in the Athabasca Oil Sands Region, Alberta. *Science of the Total Environment* **583**, 369-381 (2017).
88 Johnston, C. E. *et al.* Erratum: Effect of permafrost thaw on CO 2 and CH 4 exchange in a western Alaska peatland chronosequence (2013 Environ. Res. Lett. 9 085004). *Environmental*

Research Letters **9** (2014).
89 Trudeau, N. C., Garneau, M. & Pelletier, L. Methane fluxes from a patterned fen of the northeastern part of the La Grande river watershed, James Bay, Canada. *Biogeochemistry* **113**, 409-422 (2013).
90 Nadeau, D. F., Rousseau, A. N., Coursolle, C., Margolis, H. A. & Parlange, M. B. Summer methane fluxes from a boreal bog in northern Quebec, Canada, using eddy covariance measurements. *Atmospheric Environment* **81**, 464-474 (2013).
91 Marushchak, M. *et al.* Methane dynamics in warming tundra of Northeast European Russia. *Biogeosciences Discussions* **12** (2015).
92 Pelletier, L., Moore, T., Roulet, N., Garneau, M. & Beaulieu-Audy, V. Methane fluxes from three peatlands in the La Grande Riviere watershed, James Bay lowland, Canada. *Journal of Geophysical Research: Biogeosciences* **112** (2007).
93 Riutta, T., Korrensalo, A., Laine, A. M., Laine, J. & Tuittila, E.-S. Interacting effects of vegetation components and water level on methane dynamics in a boreal fen. *Biogeosciences* **17**, 727-740 (2020).
94 Heikkinen, J. E., Virtanen, T., Huttunen, J. T., Elsakov, V. & Martikainen, P. J. Carbon balance in East European tundra. *Global biogeochemical cycles* **18** (2004).
95 Merbold, L. *et al.* Artificial drainage and associated carbon fluxes (CO2/CH4) in a tundra ecosystem. *Global Change Biology* **15**, 2599-2614 (2009).
96 Parmentier, F. *et al.* Spatial and temporal dynamics in eddy covariance observations of methane fluxes at a tundra site in northeastern Siberia. *Journal of Geophysical Research: Biogeosciences* **116** (2011).
97 Sachs, T., Giebels, M., Boike, J. & Kutzbach, L. Environmental controls on CH4 emission from polygonal tundra on the microsite scale in the Lena river delta, Siberia. *Global Change Biology* **16**, 3096-3110 (2010).
98 Sachs, T., Wille, C., Boike, J. & Kutzbach, L. Environmental controls on ecosystem-scale CH4 emission from polygonal tundra in the Lena River Delta, Siberia. *Journal of Geophysical Research: Biogeosciences* **113** (2008).
99 Myers-Smith, I. H., McGuire, A. D., Harden, J. W. & Chapin III, F. S. Influence of disturbance on carbon exchange in a permafrost collapse and adjacent burned forest. *Journal of Geophysical Research: Biogeosciences* **112** (2007).
100 Hartley, I. P. *et al.* Quantifying landscape-level methane fluxes in subarctic Finland using a multiscale approach. *Global Change Biology* **21**, 3712-3725 (2015).
101 Pearson, M. *et al.* Effects of temperature rise and water-table-level drawdown on greenhouse gas fluxes of boreal sedge fens. (2015).
102 Dinsmore, K. J. *et al.* Growing season CH 4 and N 2 O fluxes from a subarctic landscape in northern Finland; from chamber to landscape scale. *Biogeosciences* **14**, 799-815 (2017).
103 Sabrekov, A. *et al.* Methane emissions from north and middle taiga mires of Western Siberia: Bc8 standard model. *Moscow University Soil Science Bulletin* **67**, 45-53 (2012).
104 Pirk, N. *et al.* Toward a statistical description of methane emissions from arctic wetlands. *Ambio* **46**, 70-80 (2017).
105 Ström, L., Tagesson, T., Mastepanov, M. & Christensen, T. R. Presence of Eriophorum scheuchzeri enhances substrate availability and methane emission in an Arctic wetland. *Soil Biology and Biochemistry* **45**, 61-70 (2012).

106 Korrensalo, A. *et al.* Small spatial variability in methane emission measured from a wet patterned boreal bog. *Biogeosciences* **15**, 1749-1761 (2018).

107 Öquist, M. & Svensson, B. Vascular plants as regulators of methane emissions from a subarctic mire ecosystem. *Journal of Geophysical Research: Atmospheres* **107**, ACL 10-11-ACL 10-10 (2002).

108 Malhotra, A. & Roulet, N. Environmental correlates of peatland carbon fluxes in a thawing landscape: do transitional thaw stages matter? *Biogeosciences* **12**, 3119-3130 (2015).

109 (!!! INVALID CITATION !!! 68,103,105).

110 Strack, M., Waller, M. & Waddington, J. Sedge succession and peatland methane dynamics: A potential feedback to climate change. *Ecosystems* **9**, 278-287 (2006).

111 (!!! INVALID CITATION !!! 107,108).

112 Takakai, F. *et al.* CH4 and N2O emissions from a forest-alas ecosystem in the permafrost taiga forest region, eastern Siberia, Russia. *Journal of Geophysical Research: Biogeosciences* **113** (2008).

113 Sabrekov, A., Glagolev, M., Kleptsova, I. & Maksyutov, S. Methane emission from West Siberia tundra mires. *Environmental Dynamics and Global Climate Change* **2**, 1-16 (2011).

114 Cooper, M. D. *et al.* Limited contribution of permafrost carbon to methane release from thawing peatlands. *Nature Climate Change* **7**, 507-511 (2017).

115 Van Huissteden, J., Maximov, T., Kononov, A. & Dolman, A. Summer soil CH4 emission and uptake in taiga forest near Yakutsk, Eastern Siberia. *agricultural and forest meteorology* **148**, 2006-2012 (2008).

116 Van Huissteden, J., Maximov, T. & Dolman, A. High methane flux from an arctic floodplain (Indigirka lowlands, eastern Siberia). *Journal of Geophysical Research: Biogeosciences* **110** (2005).

117 Munir, T. M. & Strack, M. Methane flux influenced by experimental water table drawdown and soil warming in a dry boreal continental bog. *Ecosystems* **17**, 1271-1285 (2014).

118 Luan, J. & Wu, J. Gross photosynthesis explains the 'artificial bias' of methane fluxes by static chamber (opaque versus transparent) at the hummocks in a boreal peatland. *Environmental Research Letters* **9**, 105005 (2014).

119 Sabrekov, A. *et al.* The spatial variability of methane emission from subtaiga and forest–steppe grass–moss fens of Western Siberia. *Biology Bulletin* **43**, 162-168 (2016).

120 Sabrekov, A., Runkle, B. R., Glagolev, M., Kleptsova, I. & Maksyutov, S. Seasonal variability as a source of uncertainty in the West Siberian regional CH4 flux upscaling. *Environmental Research Letters* **9**, 045008 (2014).

121 Wille, C., Kutzbach, L., Sachs, T., Wagner, D. & PFEIFFER, E. M. Methane emission from Siberian arctic polygonal tundra: eddy covariance measurements and modeling. *Global Change Biology* **14**, 1395-1408 (2008).

122 Zona, D. *et al.* Methane fluxes during the initiation of a large-scale water table manipulation experiment in the Alaskan Arctic tundra. *Global Biogeochemical Cycles* **23** (2009).
